# Factor analysis and GA-BP-ANN prediction of nitrogen diffusion behavior in underground laboratory under ventilation conditions

**DOI:** 10.1038/s41598-024-63829-8

**Published:** 2024-06-11

**Authors:** Baochun Li, Minghua Chi, Minglun Gao, Licong Wang, Jinlong Xu, Xiangguo Zeng

**Affiliations:** 1 Key Laboratory of Deep Underground Science and Engineering (Ministry of Education), College of Architecture and Environment, Sichuan University, Chengdu, 610065 People’s Republic of China; 2 China Construction Third Bureau Group Co., Ltd., Wuhan, 430064 People’s Republic of China

**Keywords:** Underground laboratory, Ventilation system, Nitrogen diffusion behavior, CFD technology, Latin hypercube sampling, GA-BP-ANN, Civil engineering, Environmental sciences

## Abstract

Nitrogen is widely used in various laboratories as a suppressive gas and a protective gas. Once nitrogen leaks and accumulates in a such confined space, it will bring serious threats to the experimental staff. Especially in underground tunnels or underground laboratories where there is no natural wind, the threat is more intense. In this work, the ventilation design factors and potential leakage factors are identified by taking the leakage and diffusion of a large liquid nitrogen tank in China Jinping Underground Laboratory (CJPL) as an example. Based on computational fluid dynamics (CFD) research, the effects of fresh air inlet position, fresh air velocity, exhaust outlet position, leakage hole position, leakage hole size, and leaked nitrogen mass flow rate on nitrogen diffusion behavior in specific environments are discussed in detail from the perspectives of nitrogen concentration field and nitrogen diffusion characteristics. The influencing factors are parameterized, and the Latin hypercube sampling (LHS) is used to uniformly sample within the specified range of each factor to obtain samples that can represent the whole sample space. The nitrogen concentration is measured by numerical value, and the nitrogen diffusion characteristics are measured by category. The GA-BP-ANN numerical regression and classification regression models for nitrogen concentration prediction and nitrogen diffusion characteristics prediction are established. By using various rating indicators to evaluate the performance of the trained model, it is found that models have high accuracy and recognition rate, indicating that it is effective in predicting and determining the concentration value and diffusion characteristics of nitrogen according to ventilation factors and potential leakage factors. The research results can provide a theoretical reference for the parametric design of the ventilation system.

## Introduction

Nitrogen is widely used in various laboratories because it can be used as a suppression gas and a protective gas^1,2^. Such environmental characteristics are mostly confined spaces. Once nitrogen leaks and accumulates in a confined space, it cannot be discharged in time, which will bring serious life threats to the experimental staff^3^. Although nitrogen is a non-toxic gas, excessive inhalation of nitrogen can reduce the ability of human blood to transport oxygen or tissue to use oxygen, causing organ damage and even suffocation^4^. This problem is more prominent in confined spaces, especially in underground tunnels or underground laboratories without natural wind. This puts forward higher requirements on how to design and optimize the mechanical ventilation system to better exclude nitrogen and ensure the air quality in the environment. The current research basically divides the known ventilation systems of various building types into seven ventilation modes: mixed ventilation, displacement ventilation, mixed airflow organization, stratified ventilation, occupied area protection ventilation, local scheduling ventilation, and piston ventilation^5^. Among them, mixed ventilation is to dilute the contaminated indoor air by mixing the supplied fresh air with the indoor air. Although this ventilation method is relatively rough^6^, it is widely used in the design of ventilation systems in tunnels and laboratories due to its good environmental adaptability and mature technology. Under the same ventilation mode, the effect of removing pollutants brought by different designs is also very different, which is the result of multiple factors^7^. These influencing factors can be classified into two categories: ventilation design factors and nitrogen potential leakage factors. The former includes fresh air inlet position, fresh air velocity, and exhaust outlet position, while the latter includes leakage hole position, leakage hole size, and leaked nitrogen mass flow rate. Therefore, it is of great significance to explore the diffusion behavior and characteristics of leakage nitrogen under different ventilation design factors and potential leakage factors in the deep underground laboratory to ensure the safe operation of the deep underground laboratory.

Compared with experimental research, computational fluid dynamics (CFD) shows unique advantages in studying the performance of ventilation systems^8^. A large number of studies based on CFD technology such as gas diffusion behavior^9–11^, comparison of ventilation mode applicability^12,13^, and analysis of influencing factors on ventilation system performance^14^ have appeared. In terms of gas diffusion behavior, Schmidt et al.^15^ investigated the diffusion behavior of hydrogen/air mixed clouds between buildings under meteorological conditions, release rate, release time, and release amount. Yu et al.^16^ used the mixed multiphase flow method to study the potential liquid oxygen leakage caused by tank damage or pipeline rupture and analyzed the effects of ambient temperature, leakage rate, cofferdam, and wind direction on temperature and concentration distribution. Hu et al.^17^ designed an optimized ventilation system based on CFD analysis, which solved the problem of poor ventilation efficiency in chemical plants with carbon monoxide as a pollutant. Tang et al.^18^ studied the evaporation and diffusion behavior of liquid hydrogen in open areas, garages, and tunnels, and investigated the cloud shape, duration, and hazard range of hydrogen gas cloud in different environments. Gopalaswami et al.^19^ numerically simulated the pool diffusion and vaporization of liquefied natural gas using a three-dimensional mixed homogeneous Euler multiphase solver. Kong^20^ took the actual project as an example to establish a CFD model for the leakage and diffusion of liquefied natural gas pipelines in tunnels, and analyzed the changes of CH_4_ concentration field and explosion gas cloud with time under different conditions.

In the study of influencing factors on ventilation system performance, Matsuura et al.^21^ studied the hydrogen diffusion in a partially open space with a single roof vent and discussed the effects of different roof vent positions, leakage positions, leakage flow, and exhaust flow on the forced ventilation of leaked hydrogen. Beard et al.^22^ studied the diffusion behavior of hydrogen/air mixture in a closed space and compared it with the experimental results. It is concluded that the use of the lower vent helps to dilute the hydrogen/air mixture, which in turn reduces the volume of the flammable gas cloud. Kazuo et al.^23^ studied the transient behavior and accumulation process of hydrogen in partially open space and discussed the influence of different exhaust positions and exhaust conditions on hydrogen concentration distribution. Jiao et al.^24^ evaluated the performance of a dilution ventilation system in a closed workshop environment with CO as a pollutant under seven ventilation layouts, evaluated the performance of different ventilation layouts, and obtained the optimal layout design for pollution control, which can provide a reference for improving dilution ventilation efficiency and reducing energy consumption in generally closed areas. Khan et al.^25^ found that the steady-state distribution of pollutant concentration in the workshop is a function of several factors, the most important of which is the type and relative position of the inlet and outlet. The best settings for several different inlet and outlet positions and types were determined. Han et al.^26^ studied the diffusion characteristics of SF_6_ gas in industrial buildings and the ventilation performance under the influence of leakage angle, outlet layout, and ventilation rate by combining experiments and CFD technology. Based on the CFD model, Lee et al.^27^ compared three exhaust configurations including natural ventilation and forced ventilation to deal with hydrogen leakage, and determined the relationship between exhaust port size and hydrogen concentration. In summary, CFD technology has unique advantages in the diffusion of light or heavy gases and the emission of pollutants under multi-ventilation conditions, and has become one of the main research methods for studying gas flow problems. The above research objectives involve a variety of gas-phase or liquid-phase pollutants, and CFD technology is applicable enough. Therefore, this work uses CFD technology and carries out its accuracy verification to ensure its accuracy in studying the diffusion behavior of nitrogen under different ventilation conditions.

In addition, there are many factors that affect the removal efficiency of pollutants in the ventilation system. The interaction between factors makes the prediction of pollutant concentration and distribution complicated. To get rid of this dilemma, researchers have introduced the concept of machine learning into the ventilation system optimization design, providing relevant experience that can accurately predict the concentration and distribution of pollutants. Machine learning technology has unique advantages in dealing with nonlinear problems. In this regard, Zhang et al.^28^ used genetic algorithm (GA) as an enhancement method to improve the global search ability of back propagation artificial neural network (BP-ANN), and established a GA-BP-ANN model for predicting rolling force of ultra-thick plate with high accuracy, and the prediction error was reduced to 3.95%. Ren et al.^29^ introduced a BP-ANN based on particle swarm optimization and applied it to the prediction of wind speed. Based on the comparison of actual wind speed data, it has good prediction accuracy. Yu et al.^30^ proposed a short-term gas load forecasting method based on an improved BP-ANN model, which can obtain a more ideal short-term gas load forecasting solution. In terms of ventilation technology, Kim et al.^31^ used an ANN algorithm to predict and evaluate the influence of mechanical ventilation rate, natural ventilation rate, and local weather conditions on the actual ventilation performance of residential buildings, which proved the applicability and accuracy of ANN algorithm in predicting ventilation performance. Park et al.^32^ used the ANN algorithm to predict the particulate matter concentration in subway stations by combining particulate matter information, subway operation frequency information, and ventilation operation information and concluded that there is a high correlation between the predicted value and the measured value of the artificial neural network model. Ren et al.^33^ realized the online control of indoor air quality by combining a low-dimensional linear ventilation model and an ANN algorithm. The combination of machine learning technology and CFD technology can provide a strategy to reduce the difficulty and cost of ventilation system optimization design. Li et al.^34^ proposed an algorithm combining a BP-ANN model based on CFD technology and an adaptive multi-objective particle swarm optimizer to predict and control the concentration of CO_2_ and PM2.5. Zhang et al.^35^ took fresh air parameters such as air supply volume, air inlet temperature, and air inlet angle as design variables, and took thermal comfort as a restrictive design goal. An optimization method combining GA, ANN algorithm, multiple regression analysis, and fuzzy logic controller was proposed to optimize indoor air quality and energy consumption. It is not difficult to see that machine learning technology has given great help to the optimization design of ventilation systems and provided strong convenience.

In this work, CFD research is carried out based on the possible nitrogen leakage and diffusion problems faced by large liquid nitrogen tanks in China Jinping Underground Laboratory (CJPL). The effects of fresh air inlet position, fresh air velocity, exhaust outlet position, leakage hole position, leakage hole size, and nitrogen mass flow rate on nitrogen diffusion behavior and characteristics in a specified environment are studied. Based on the numerical regression and classification regression techniques of the BP neural network machine learning model optimized by GA, combined with Latin hypercube sampling (LHS), the prediction models of nitrogen concentration and nitrogen diffusion characteristics with good generalization ability and multi-factor and multi-range prediction ability are built and trained respectively. The research results can provide a theoretical reference for the parametric design of the ventilation system.

## Methods

### Research problem

**Figure 1:**
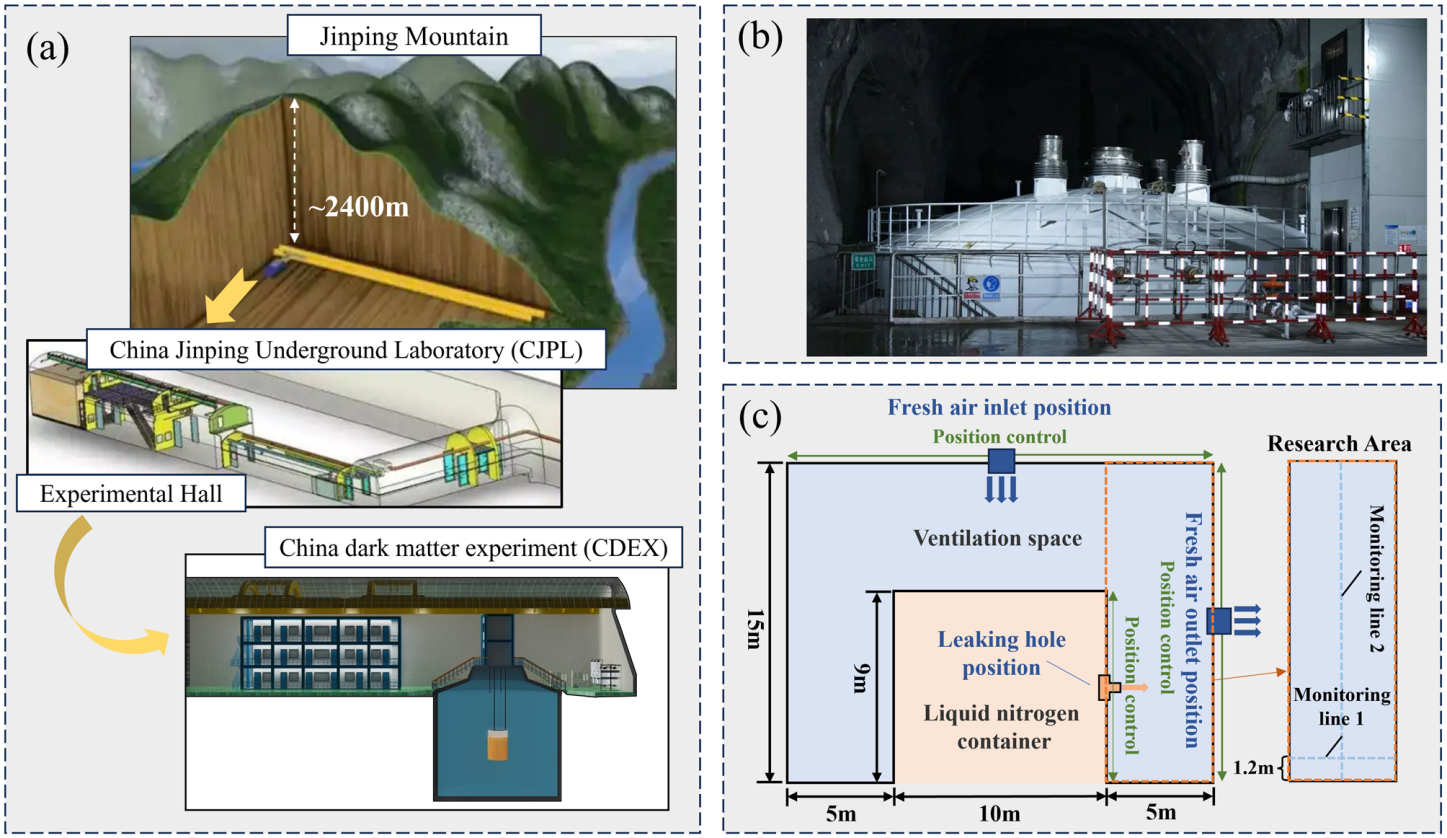
Research question: (**a**) schematic diagram of the Jinping Underground Laboratory, (**b**) liquid nitrogen tank, and (**c**) simplified 2D structural diagram used in this work.

As the world’s deepest laboratory, the “China Jinping Underground Laboratory (CJPL)” is located inside Jinping Mountain, with a mountain cover of about 2400 m, as shown in Fig. 1a. The cosmic ray flux inside the laboratory is only one hundred millionth of the surface. Based on this feature, it carries the dark matter detection experiment, which requires a large liquid nitrogen tank as a shielding facility, as shown in Fig. 1b. In the deep laboratory without natural wind, the only measure to prevent nitrogen leakage is to design a reasonable ventilation system. According to the actual situation of the experimental hall, the experimental hall is simplified to a 2D model of its longitudinal section, as shown in Fig. 1c. The size of the longitudinal section of the experimental hall is 20 × 15 m, and the size of the liquid nitrogen tank is 10 × 9 m. According to the actual design of the ventilation system, the fresh air inlet is only set on the top of the hall, and the exhaust port is set on the side wall of the hall. The length of the fresh air inlet and exhaust port is 1 m.

In order to explore the influence of different factors on the diffusion of leaked nitrogen, this study considers two factors: the ventilation system design factor and the potential nitrogen leakage factor. Ventilation system design factors include fresh air inlet position (IP), fresh air inlet velocity (IV), and exhaust outlet position (OP). The potential leakage factors include leakage hole position (LP), leakage hole size (LS), and nitrogen mass flow rate (NM). The research condition design is shown in Table 1. The research objectives focus on the nitrogen concentration distribution in the research area (the area surrounded by orange lines) shown in Fig. 1c, the change of nitrogen concentration on horizontal monitoring line 1 (1.2 m from the ground is the personnel breathing line) and the change of nitrogen concentration on vertical monitoring line 2. By observing the nitrogen concentration distribution on monitoring line 1, the nitrogen removal efficiency can be evaluated from the perspective of human respiratory safety, while the diffusion height of nitrogen can be roughly obtained by observing the nitrogen concentration distribution on monitoring line 2. In this work, the complex engineering problems are simplified: (1) Without considering the phase transition process of liquid nitrogen, only the gaseous diffusion is considered. (2) The nitrogen concentration only refers to the leakaged nitrogen, excluding the original nitrogen content in the air. These two simplifications aim to remove the complexity and focus on the influence of different factors on nitrogen diffusion behavior.

**Table 1:** Single factor design.

Classification	Factor	Quantitative value
Ventilation design factors	Horizontal distance between fresh air inlet and nitrogen leak hole- IP (m)	− 5/− 2.5/0/2.5/5/7.5/10/12.5/15
	Fresh air velocity-IV (m/s)	0.1/0.25/0.5/1/2/3/5
	Height of exhaust outlet-OP (m)	0.5/2.5/4.5/6.5/8.5/10.5/12.5/14.5
Potential leakage factors	Height of leakage hole-LP (m)	0/1.5/3/4.5/6/7.5/9
	Leakage size-LS (mm)	10/50/100/200/300/400/500
	Nitrogen mass flow rate-NM (kg/s)	0.1/0.25/0.5/1/2/3/5

### Nitrogen diffusion simulation

#### Mathematical model

The nitrogen diffusion in the ventilated state obeys the basic control equation, transport equation, and ideal gas state equation^36^. The mathematical models are shown in Table 2.

**Table 2:** Governing equations of fluid.

Conservation of mass^37^:	$\frac{\partial }{{\partial x_{i} }}\left( {\rho u_{i} } \right) = 0$
Conservation of momentum^37^:	$\frac{\partial }{{\partial x_{j} }}\left( {\rho u_{i} u_{j} } \right) = - \frac{\partial p}{{\partial x_{i} }} + \frac{{\partial \overline{{\overline{{\tau_{ij} }} }} }}{{\partial x_{j} }} + \frac{\partial }{{\partial x_{j} }}\left( { - \rho \overline{{u_{i}^{\prime } u_{j}^{\prime } }} } \right) + \rho g_{i} + F_{i}$
Conservation of energy^38^:	$\frac{\partial }{{\partial x_{i} }}\left[ {u_{i} (\rho E + p)} \right] = \frac{\partial }{{\partial x_{j} }}\left[ {k_{eff} \frac{\partial T}{{\partial x_{j} }} + u_{i} \left( {\overline{{\overline{{\tau_{ij} }} }} } \right)_{eff} } \right]$
Transport equation^39^:	$\frac{\partial }{\partial t}(\rho \omega ) + \frac{\partial }{{\partial x_{j} }}\left( {\rho u_{j} \omega } \right) = \frac{\partial }{{\partial x_{j} }}\left( {u_{i} \frac{\partial \omega }{{\partial x_{j} }}} \right)$
	$$\rho = \frac{p}{RT}\frac{{M_{v} M_{a} }}{{\left[ {\omega M_{a} + (1 - \omega )M_{v} } \right]}}$$
Equation of state^40^:	$pv = RT$

The standard $k - \varepsilon$ model is selected as the turbulence model^41,42^. This model assumes that the flow is completely turbulent, and the influence of molecular viscosity can be ignored. The correlation equations are shown in Table 3, and the relevant empirical constants are shown in Table 4^43^.

**Table 3:** Standard $k - \varepsilon$ turbulence model.

$$\frac{\partial }{{\partial x_{i} }}\left( {\rho \kappa u_{i} } \right) = \frac{\partial }{{\partial x_{j} }}\left[ {\left( {\mu + \frac{{\mu_{t} }}{{\sigma_{\kappa } }}} \right)\frac{\partial \kappa }{{\partial x_{j} }}} \right] + G_{\kappa } + G_{b} + Y_{M} - \rho \varepsilon$$
$$\frac{\partial }{{\partial x_{i} }}\left( {\rho \varepsilon u_{i} } \right) = \frac{\partial }{{\partial x_{j} }}\left[ {\left( {\mu + \frac{{\mu_{t} }}{{\sigma_{\varepsilon } }}} \right)\frac{\partial \varepsilon }{{\partial x_{j} }}} \right] + C_{1\varepsilon } \frac{\varepsilon }{\kappa }G_{\kappa } - C_{2\varepsilon } \rho \frac{{\varepsilon^{2} }}{\kappa }$$
$$G_{\kappa } = 2\mu_{t} \left[ {\left( {\frac{\partial u}{{\partial x}}} \right)^{2} + \left( {\frac{\partial v}{{\partial y}}} \right)^{2} + \left( {\frac{\partial w}{{\partial z}}} \right)^{2} } \right] + \left( {\frac{\partial u}{{\partial y}} + \frac{\partial v}{{\partial x}}} \right)^{2} + \left( {\frac{\partial u}{{\partial z}} + \frac{\partial w}{{\partial x}}} \right)^{2} + \left( {\frac{\partial w}{{\partial y}} + \frac{\partial v}{{\partial z}}} \right)^{2}$$
$$G_{b} = - g_{i} \frac{{\mu_{t} }}{{\rho \Pr_{t} }}\frac{\partial \rho }{{\partial x_{i} }}$$
$$Y_{M} = 2\rho \varepsilon \frac{\kappa }{\gamma RT}$$
$$\mu_{t} = \rho C_{\mu } \frac{{\kappa^{2} }}{\varepsilon }$$

**Table 4:** Correlation constants of $k - \varepsilon$ turbulence model^44^.

Constant	$C_{1\varepsilon }$	$C_{2\varepsilon }$	$\sigma_{\kappa }$	$\sigma_{\varepsilon }$	$\Pr_{t}$	$C_{\mu }$
Value	1.44	1.92	1.0	1.3	0.85	0.09

#### Numerical simulation

In this study, the finite volume method of ANSYS/FLUENT software is used to solve the problem, and the control equation is discretized based on the pressure solver. The concentration distribution of leakage nitrogen under the influence of different factors is studied by the steady-state simulation method. According to the working conditions shown in Table 1, the corresponding two-dimensional numerical model is established one by one. The boundary conditions are shown in Table 5.

**Table 5:** Definition of boundary type.

Boundary location	Boundary type
Ventilation inlet	Velocity inlet
Ventilation outlet	Pressure outlet
Leak hole	Mass flow inlet
Other walls	Wall

**Figure 2:**
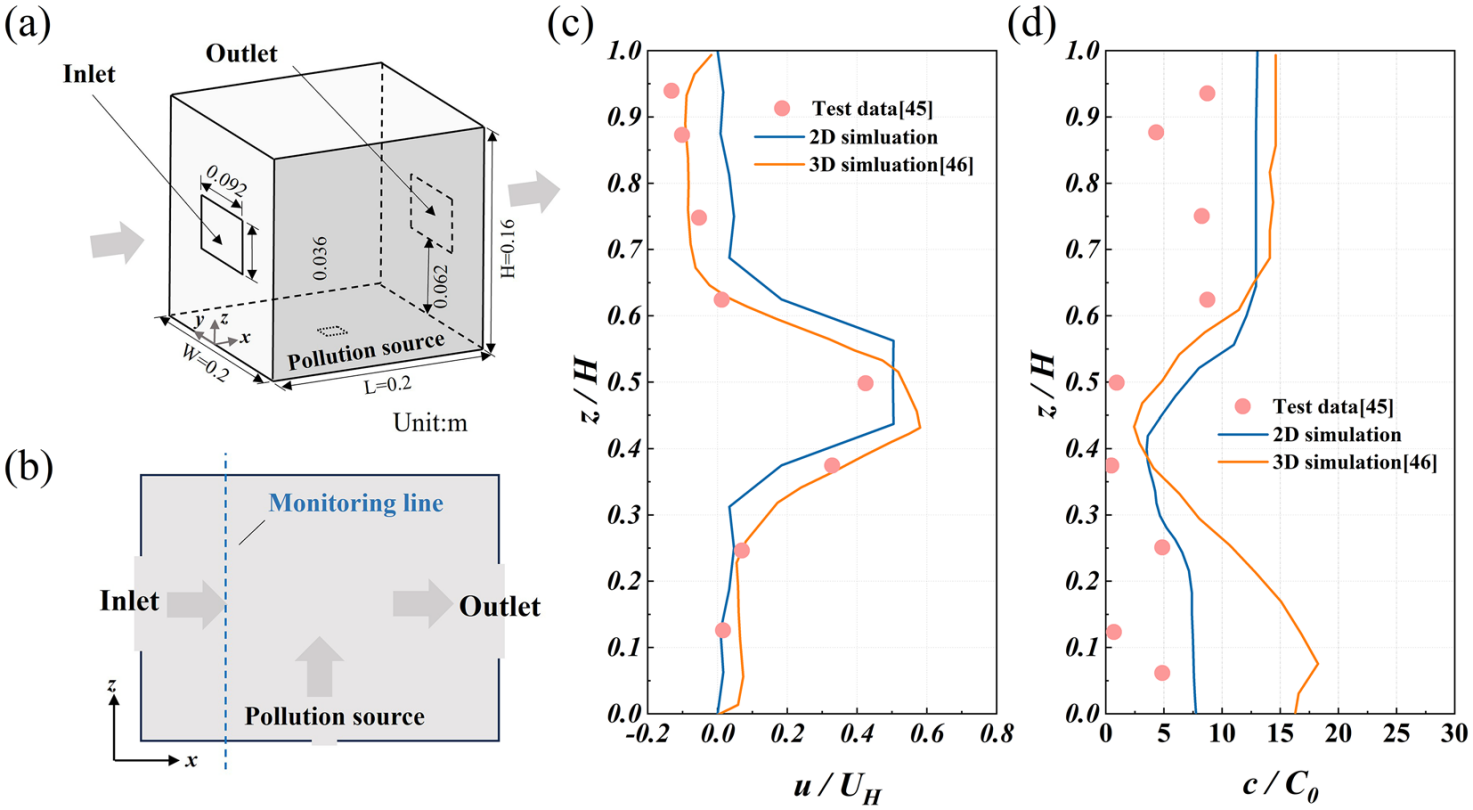
Model accuracy verification: (**a**) 3D schematic diagram of the building model, (**b**) 2D schematic diagram of the building model, (**c**) a comparison of wind speed on the monitoring line, and (**d**) a comparison of pollutant concentration on the monitoring line.

The wind tunnel test data given by Tominaga et al.^45^ and the 3D numerical simulation data given by Xie et al.^46^ are used to verify the accuracy of the method such as steady-state simulation and 2D model used in this study. Figure 2a is a schematic diagram of the building model based on the numerical simulation given by the wind tunnel test. The ratio of the building model to the prototype is 1:100. The 3D model is simplified into a 2D model with y = 0 for numerical simulation, and the speed and concentration monitoring lines are set, as shown in Fig. 2b. Figure 2c is a comparison of wind speed on the monitoring line, and Fig. 2d is a comparison of pollutant concentration on the monitoring line. The inlet wind speed is 0.5 *U*_*H*_, and the *U*_*H*_ is 4.3 m/s. The pollutant is a mixture of C_2_H_4_ and air. The pollution source is a point source, which is located in the center of the ground in the model. The size of the release port is 8 mm × 8 mm, and the volume fraction of C_2_H_4_ is 5 × 10^−3^. To facilitate the comparison, the C_2_H_4_ dimensionless concentration *c/C*_0_ was defined, where *c* is the volume fraction of C_2_H_4_, *C*_0_ = *q*e/(*H*^*2*^*U*_*H*_), where *q*e is the release rate, and the unit is m^3^/s. It can be seen from Fig. 2c and d that the results obtained by the 2D model and the steady-state numerical simulation are in good agreement with the experimental data in terms of both the value and distribution trend of wind speed and concentration. Therefore, the theory adopted in this study is reasonable and accurate.

**Figure 3:**
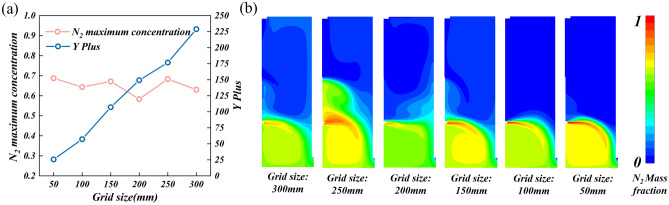
Verification of grid size: (**a**) Yplus on the wall surface and the maximum nitrogen concentration on monitoring line 1 under different grid sizes, and (**b**) nitrogen concentration distribution cloud map in the research area under different grid sizes.

In terms of grid size, the independence verification and Yplus verification of grid size are carried out. Refining the grid can increase the computational cost while improving the computational accuracy^47^. It is necessary to find a reasonable grid size so that the continuous refinement of the grid will cause little change to the calculation results. The maximum concentration of leakage nitrogen on monitoring line 1 is selected as the basis for judgment. Taking the research conditions of fresh air inlet position IP = 0 m, fresh air velocity IV = 0.5 m/s, exhaust outlet position OP = 0.5 m, leakage hole position LP = 4.5 m, leakage hole size LS = 200 mm, and nitrogen mass flow rate NM = 0.5 kg/s as examples, six different grid sizes of 50, 100, 150, 200, 250, and 300 mm are selected to compare the maximum concentration of leakage nitrogen and the concentration distribution of leakage nitrogen on monitoring line 1 under different grid sizes, as shown in Fig. 3. When the grid size is 100 mm, the nitrogen concentration fluctuates less and the concentration distribution gradually tends to be consistent when the grid is further refined. It can be considered that the threshold of the grid-independent solution has been reached. Due to the use of the standard $k - \varepsilon$ turbulence model and standard wall function, the influence of the wall Yplus should also be considered. By comparing the wall Yplus under six grid sizes, it is found that when the grid size is 100 mm, the Yplus value is about 50, which meets the Yplus requirements while satisfying the grid independence. Therefore, a grid size of 100 mm is selected in this work. In addition, the grids near the leakage hole and the fresh air inlet are locally refined.

### GA-BP neural network

#### BP neural network

**Figure 4:**
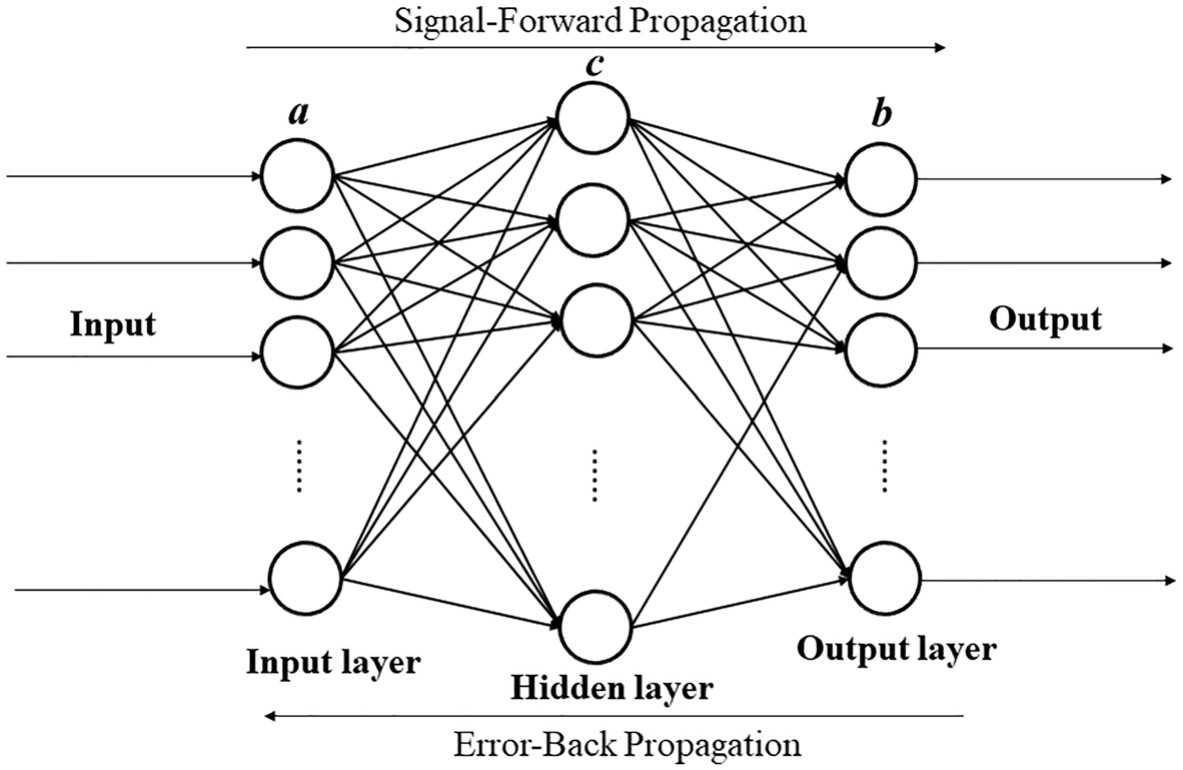
Three-layer BP neural network structure.

BP neural network is a multi-layer feedforward neural network trained by an error backpropagation algorithm. BP neural network includes an input layer, hidden layer, and output layer. The simple three-layer BP neural network structure is shown in Fig. 4a is the number of neurons in the input layer, c is the number of neurons in the hidden layer, and b is the number of neurons in the output layer.

The signal forward propagation and the error backpropagation are the main learning processes of the BP neural network. The signal forward propagation is to judge whether the algorithm continues by comparing the output error with the expected error. If the output error is less than the expected error, the algorithm ends and outputs the result. If the output error is greater than the expected error, the error of each node of the network is calculated reversely layer by layer, the weights and thresholds between layers are adjusted, and then the input is readjusted, and the forward propagation is carried out until the expected error is reached. Table 6 lists the relevant calculation formulas of BP neural network signal forward propagation and error back propagation.

**Table 6:** Related formulas of BP neural network signal forward propagation and error back propagation.

Forward propagation	
Input of hidden layer:	$A_{m} = \mathop \sum \limits_{m = 1}^{a} \left( {\varphi_{mn} x_{m} } \right) + b_{q}$
Output of hidden layer:	$Y_{m} { = }f_{1} (A_{m} )$
Input value of the output layer:	$S_{q} = \mathop \sum \limits_{m = 1}^{c} \varphi_{qm} Y_{m} + a_{q}$
Output value of the output layer:	$Q_{q} { = }f_{2} (S_{q} )$
Mean square error (MSE) between the output value and the expected value of single layer:	$MSE_{P} = \frac{1}{2}\mathop \sum \limits_{q = 1}^{b} \left( {P_{q} - Q_{q} } \right)^{2}$
Total error of all training samples:	$MSE = \frac{1}{2}\mathop \sum \limits_{c = 1}^{N} \mathop \sum \limits_{q = 1}^{b} \left( {P_{q} - Q_{q} } \right)^{2}$
Back propagation adjustment	
Hidden layer weight change:	${\Delta }\varphi_{mn} = - \eta \frac{\partial MSE}{{\partial \varphi_{mn} }} = - \eta \frac{\partial MSE}{{\partial A_{m} }}\frac{{\partial A_{m} }}{{\partial \varphi_{mn} }} = - \eta \frac{\partial MSE}{{\partial Y_{m} }}\frac{{\partial Y_{m} }}{{\partial A_{m} }}\frac{{\partial A_{m} }}{{\partial \varphi_{mn} }}$
Hidden layer threshold change:	${\Delta }b_{q} = - \eta \frac{\partial MSE}{{\partial b_{q} }} = - \eta \frac{\partial MSE}{{\partial A_{m} }}\frac{{\partial A_{m} }}{{\partial MSEb_{q} }} = - \eta \frac{\partial MSE}{{\partial Y_{m} }}\frac{{\partial Y_{m} }}{{\partial A_{m} }}\frac{{\partial A_{m} }}{{\partial b_{q} }}$
Output layer weight change:	${\Delta }\varphi_{qm} = - \eta \frac{\partial MSE}{{\partial \varphi_{qm} }} = - \eta \frac{\partial MSE}{{\partial S_{q} }}\frac{{\partial S_{q} }}{{\partial \varphi_{qm} }} = - \eta \frac{\partial MSE}{{\partial Q_{q} }}\frac{{\partial Q_{q} }}{{\partial S_{q} }}\frac{{\partial S_{q} }}{{\partial \varphi_{qm} }}$
Output layer threshold change:	${\Delta }a_{q} = - \eta \frac{\partial MSE}{{\partial a_{q} }} = - \eta \frac{\partial MSE}{{\partial S_{q} }}\frac{{\partial S_{q} }}{{\partial a_{q} }} = - \eta \frac{\partial MSE}{{\partial Q_{q} }}\frac{{\partial Q_{q} }}{{\partial S_{q} }}\frac{{\partial S_{q} }}{{\partial a_{q} }}$
Adjusted hidden layer weights:	$\varphi_{mn}^{\prime } = \varphi_{mn} + {\Delta }\varphi_{mn}$
Adjusted hidden layer threshold:	$b_{q}^{\prime } = b_{q} + {\Delta }b_{q}$
Adjusted output layer weights:	$\varphi_{qm}^{\prime } = \varphi_{qm} + {\Delta }\varphi_{qm}$
Adjusted output layer threshold:	$a_{q}^{\prime } = a_{q} + {\Delta }a_{q}$

#### Genetic Algorithm

**Figure 5:**
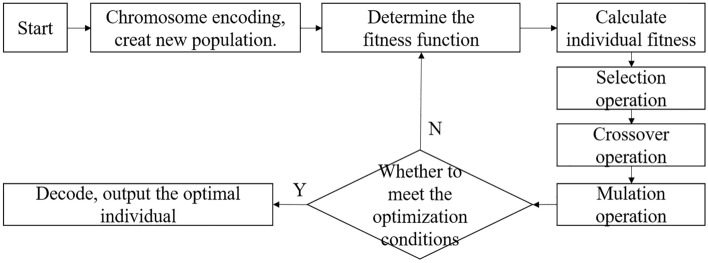
The operation process of genetic algorithm.

Genetic Algorithm (GA) is an adaptive probability optimization algorithm that combines Darwin's evolution theory with Mendel’s genetics principle and is suitable for complex system optimization. The basic principle is: based on the fitness function to carry out selection, crossover, and mutation screening of individuals, and retain the offspring with high fitness. GA has many advantages, making it widely used in many practical problems such as function optimization, neural networks, image recognition, and optimal scheduling^48^. The GA operation process is shown in Fig. 5^49^.

#### Using GA to optimize the BP neural network

**Figure 6:**
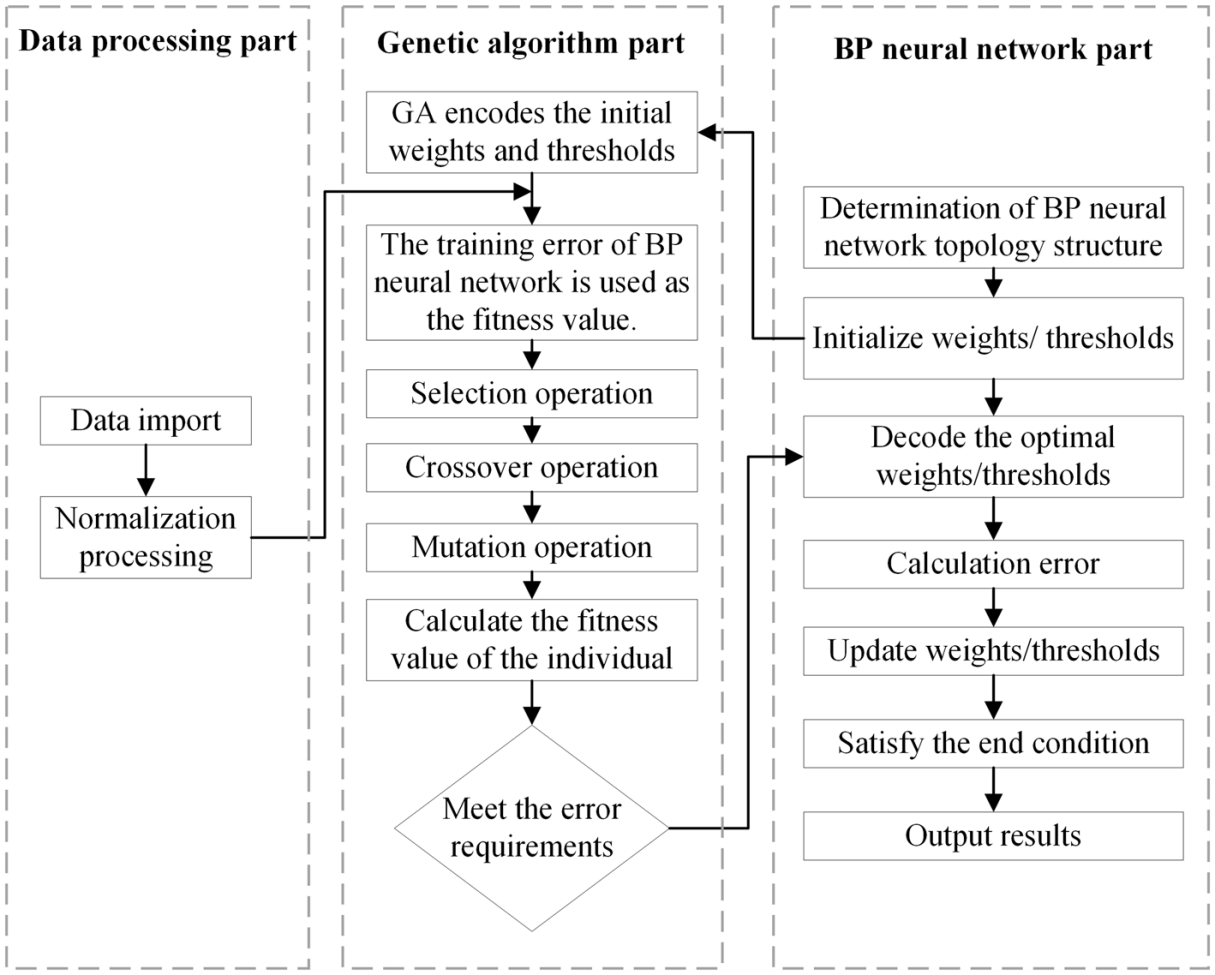
Flow chart of the GA-BP-ANN.

The selection of weights and thresholds has a great influence on the accuracy of BP neural network prediction results. If these two parameters are improperly selected, the model will easily fall into the local optimum. The universality of the BP neural network can be improved by embedding GA into the BP neural network. Firstly, the neural network structure is constructed, and then the weights and thresholds of the neural network are iterated and corrected by the GA method. Finally, the weights and thresholds in the original structure are replaced by the corrected weights and thresholds for prediction. The operation process of the GA-BP neural network is shown in Fig. 6.

## Parametric study on the influencing factors of nitrogen diffusion behavior.

### Ventilation design factors

#### Inlet position of fresh air

**Figure 7:**
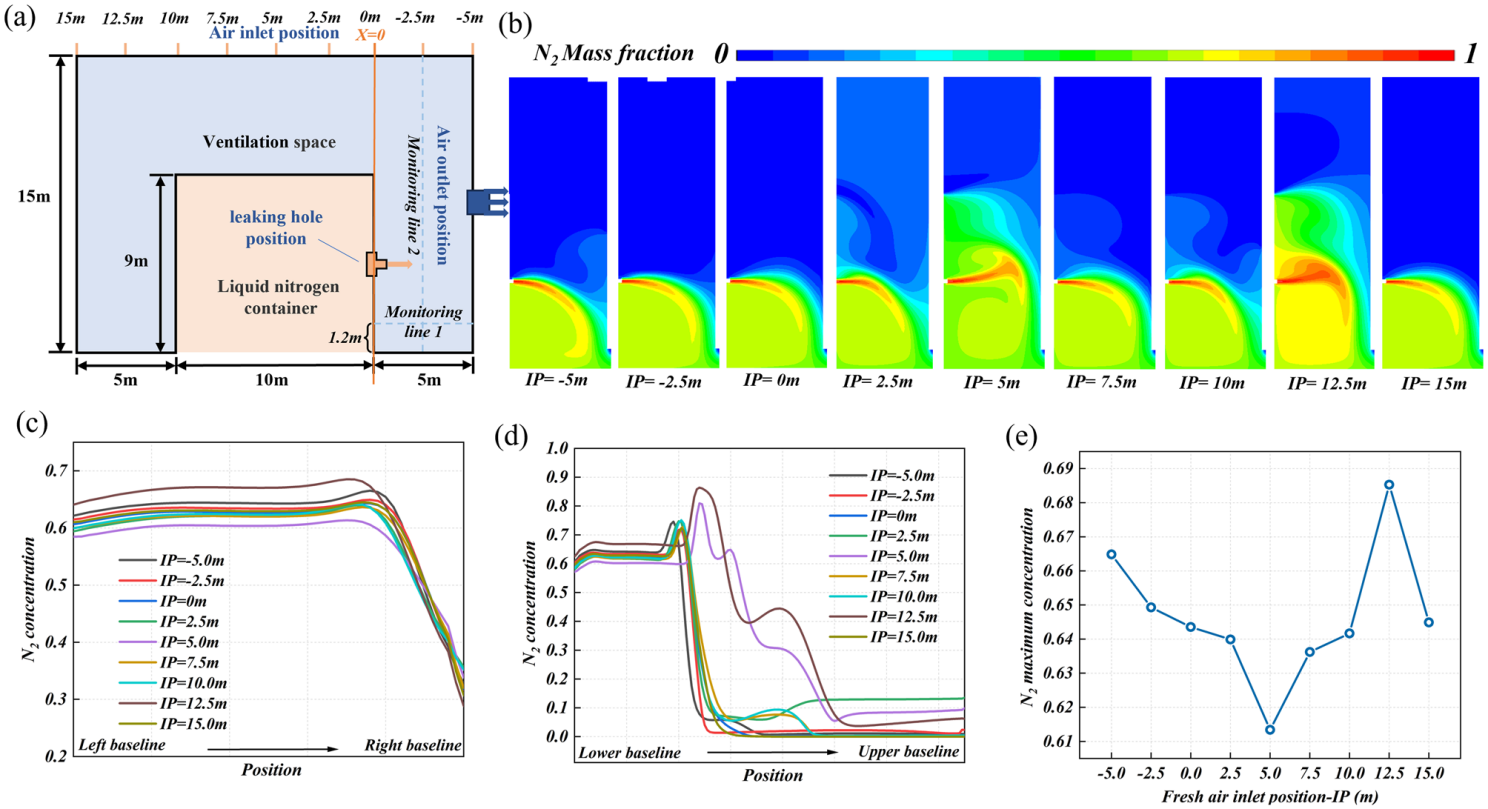
The nitrogen diffusion behavior at different fresh air inlet positions: (**a**) the work condition diagram, (**b**) the concentration distribution cloud diagram of leakage nitrogen in the research area, (**c**) the nitrogen concentration distribution on monitoring line 1, (**d**) the nitrogen concentration distribution on monitoring line 2, and (**e**) the maximum nitrogen concentration on monitoring line 1.

Fresh air inlet position has an important influence on the nitrogen diffusion behavior. The vertical line where the potential leak hole is located (left baseline of the research area) is set to IP = 0, as shown in Fig. 7a. Fresh air inlet position is parameterized as the distance from this baseline, to explore the influence of the fresh air inlet position on the leakage and diffusion of nitrogen. Figure 7b shows the nitrogen concentration distribution in the research area under different fresh air inlet positions. Under the action of fresh air flow, the flow direction of nitrogen gradually becomes consistent with that of fresh air. When the fresh air inlet and the nitrogen leakage hole are located on the same side (IP < 0), this effect is most obvious, and the nitrogen distribution is more concentrated. This is due to the air curtain formed by the fresh air flow, which plays a role in blocking nitrogen diffusion so that nitrogen diffusion is suppressed. By comparing the three working conditions of IP = − 5 m, IP = − 2.5 m, and IP = 0 m, it can be seen that the closer the fresh air inlet position is to the position of IP = 0, the larger the coverage of the air curtain, the stronger the inhibition of nitrogen diffusion. When the fresh air inlet and the leakage hole are not on the same side (IP > 0), there will be an obstacle (tank) between the fresh air inlet and the exhaust port, and the fresh air flow will flow around and the flow field will change. With the change of the fresh air inlet, the flow field of the fresh air flow will continue to change, making the nitrogen distribution area fluctuate greatly.

Figure 7c and d show the distribution of nitrogen concentration on horizontal monitoring line 1 and vertical monitoring line 2 under different fresh air inlet positions. The concentration changes on monitoring line 1 are similar. The concentration near the potential leakage hole is high, about 60%, and the nitrogen concentration near the exhaust outlet decreases sharply. Therefore, it is necessary to pay more attention to the distribution area of leakage nitrogen and pursue a smaller distribution area. The distribution area of nitrogen can be simply represented by the concentration change on monitoring line 2. Figure 7d shows that the high concentration area is also mainly concentrated in the lower part of the research area. However, when IP > 0 m, the concentration distribution on monitoring line 2 fluctuates greatly, which also indicates that nitrogen has some upward diffusion, especially when IP = 5 m and 12.5 m, the distribution area of nitrogen increases obviously, which can also be confirmed by Fig. 7b. Figure 7e is the maximum nitrogen concentration comparison on the horizontal monitoring line 1 under different fresh air inlet positions. As the inlet shifts to the potential leakage hole, the concentration decreases. Although the maximum nitrogen concentration on monitoring line 1 is the smallest when IP = 5 m, the nitrogen distribution area is larger at this time, which is not the best choice. There is no doubt that from the perspective of nitrogen concentration or nitrogen diffusion area, the nitrogen removal effect is the best when the potential leakage hole and the fresh air inlet are on the same side. Reducing the distance between the fresh air inlet and the potential leakage hole is also a powerful means to control the pollutant removal effect of the ventilation system.

#### Velocity of fresh air

**Figure 8:**
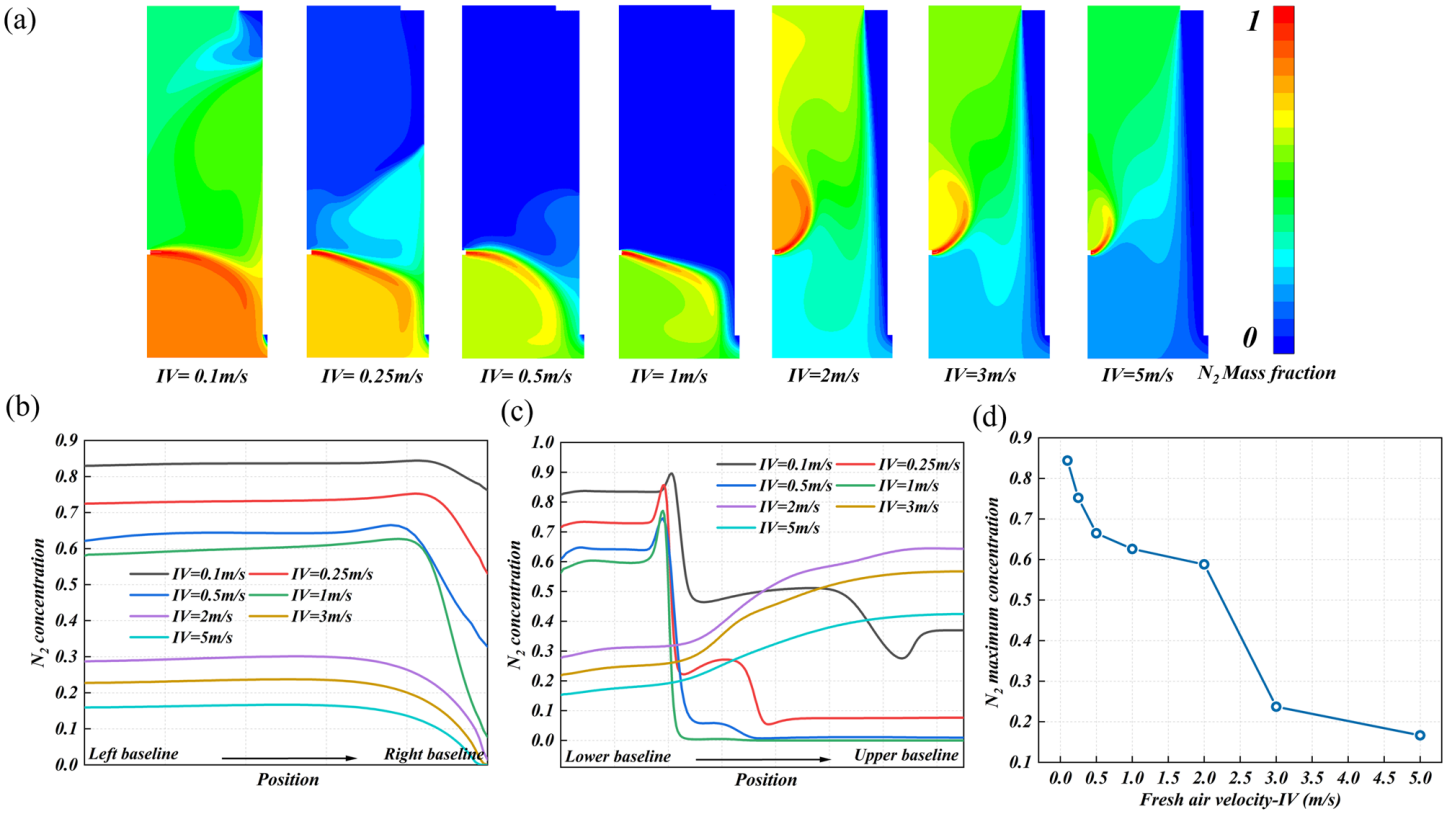
The nitrogen diffusion behavior at different f Fresh air velocities: (**a**) the concentration distribution cloud diagram of leakage nitrogen in the research area, (**b**) the nitrogen concentration distribution on monitoring line 1, (**c**) the nitrogen concentration distribution on monitoring line 2, and (**d**) the maximum nitrogen concentration on monitoring line 1.

The fresh air velocity is a manifestation of the fan frequency, which is undoubtedly closely related to the nitrogen flow field and concentration distribution. Figure 8a shows the nitrogen concentration distribution in the research area under different fresh air velocities. When IV = 0.1 and 0.25 m/s, due to the small velocity of fresh air, the blocking effect of the air curtain is not obvious, and the nitrogen distribution area is large. When the speed increases to IV = 0.5 and 1 m/s, the blocking effect of the fresh air curtain on nitrogen is obviously enhanced, and the nitrogen diffusion area gradually becomes smaller. According to this trend, it seems that the increase in fresh air velocity has a positive effect on the nitrogen removal effect. However, this is not the case. When the fresh air velocity increases to IV = 2 m/s, the blocking effect of the fresh air curtain is further strengthened. The air curtain blocks the exhaust outlet so that the nitrogen cannot be discharged smoothly. Instead, the nitrogen diffuses away from the exhaust outlet. Too high fresh air velocity weakens the effect of nitrogen removal, and this negative effect will continue to increase with the increase of fresh air velocity (IV = 3 and 5 m/s). Therefore, the control of fresh air velocity is important in the ventilation design.

Figure 8b and c show the nitrogen concentration distribution on monitoring line 1 and monitoring line 2 under different fresh air velocities. The trend of concentration change on monitoring line 1 is similar. The concentration is high in the area near the potential leakage hole and decreases sharply in the area near the exhaust outlet. The difference lies in the magnitude of the concentration value, which can be confirmed by Fig. 8d. With the increase of fresh air velocity, the maximum nitrogen concentration on monitoring line 1 decreases rapidly. However, this decreasing trend does not mean that the nitrogen removal efficiency is enhanced. When IV > 2 m, the nitrogen diffusion path changes and is not effectively discharged. Only in the appropriate fresh air velocity range (IV = 0.1–1 m/s), it can be concluded that the nitrogen removal efficiency increases with the increase of fresh air velocity, and the maximum nitrogen concentration decreases from 85% at IV = 0.1 m/s to 62% at IV = 1 m/s. This can also be confirmed by Fig. 8c. When IV < 2 m/s, the distribution trend of nitrogen is similar, and the ventilation design is effective. Among them, when IV = 0.1 and 0.25 m/s, the nitrogen concentration still shows significant fluctuations after the first sudden drop and does not decrease to 0, indicating a high nitrogen diffusion height. When IV = 0.5 and 1 m/s, the nitrogen concentration drops to 0 after the first sudden drop, indicating a smaller nitrogen diffusion area. When IV > 2 m/s, the nitrogen concentration distribution is the same, showing a slow upward trend, and the ventilation design is invalid.

#### Position of exhaust outlet

**Figure 9:**
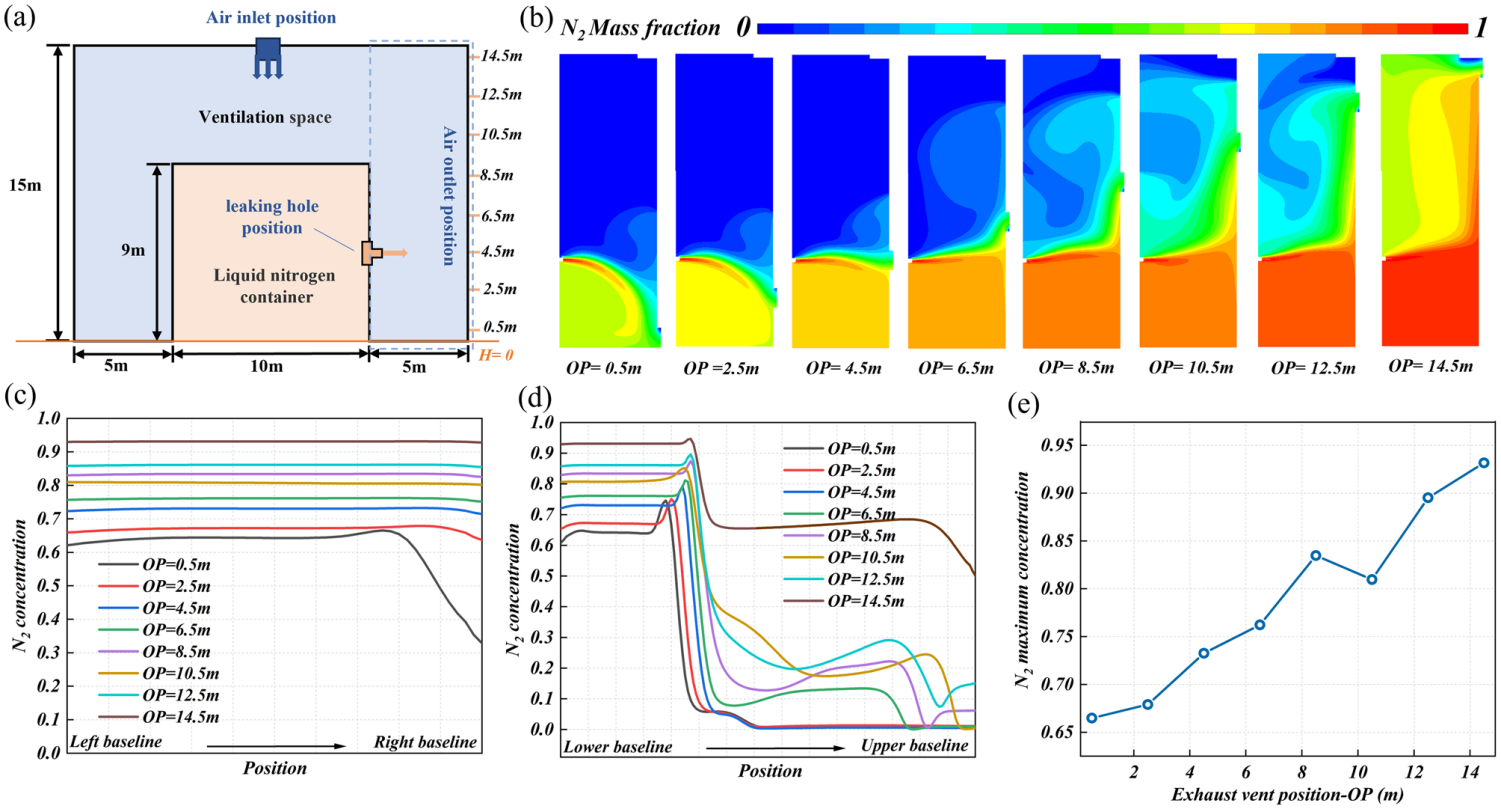
The nitrogen diffusion behavior at different exhaust outlet positions: (**a**) the work condition diagram, (**b**) the concentration distribution cloud diagram of leakage nitrogen in the research area, (**c**) the nitrogen concentration distribution on monitoring line 1, (**d**) the nitrogen concentration distribution on monitoring line 2, and (**e**) the maximum nitrogen concentration on monitoring line 1.

The location of the exhaust outlet is a ventilation design factor, which is closely related to the nitrogen flow field and concentration distribution. The ground is set to baseline OP = 0, as shown in Fig. 9a. The exhaust outlet position is parameterized as the distance from the baseline, to explore the influence of the exhaust outlet position on the nitrogen diffusion. Figure 9b shows the nitrogen concentration distribution in the research area under different exhaust outlet positions. The increase in the height of the exhaust outlet increases the diffusion distance of the leakage nitrogen, which makes the nitrogen diffusion area diverge obviously. Figure 9c and d show the nitrogen concentration distribution on monitoring line 1 and monitoring line 2 under different exhaust outlet positions. It can be seen from Fig. 9c that only when OP = 0.5 m, the nitrogen concentration on monitoring line 1 decrease sharply near the right baseline, from about 64% to about 33%. When other exhaust outlets are arranged, the nitrogen concentration distribution on monitoring line 1 is similar, and the concentration of each point is almost the same. The difference is only the nitrogen concentration. As the height of the exhaust outlet increases, the nitrogen concentration increases from 67% at OP = 2.5 m to 93% at OP = 14.5 m (Fig. 9e). It can be seen from Fig. 9d that when OP is 4.5 m, the upper concentration fluctuation on monitoring line 2 becomes larger, which means that the nitrogen diffusion area becomes larger and diverges upward. It is most obvious when OP = 14.5 m. The maximum value of nitrogen concentration on monitoring line 2 will also gradually increase with the increase of the exhaust outlet height, from 74% at OP = 0.5 m to 95% at OP = 14.5 m. Various evidence shows that the high position of the exhaust outlet is not conducive to improving the effect of nitrogen removal.

### Potential leakage factors

#### Position of leakage hole

**Figure 10:**
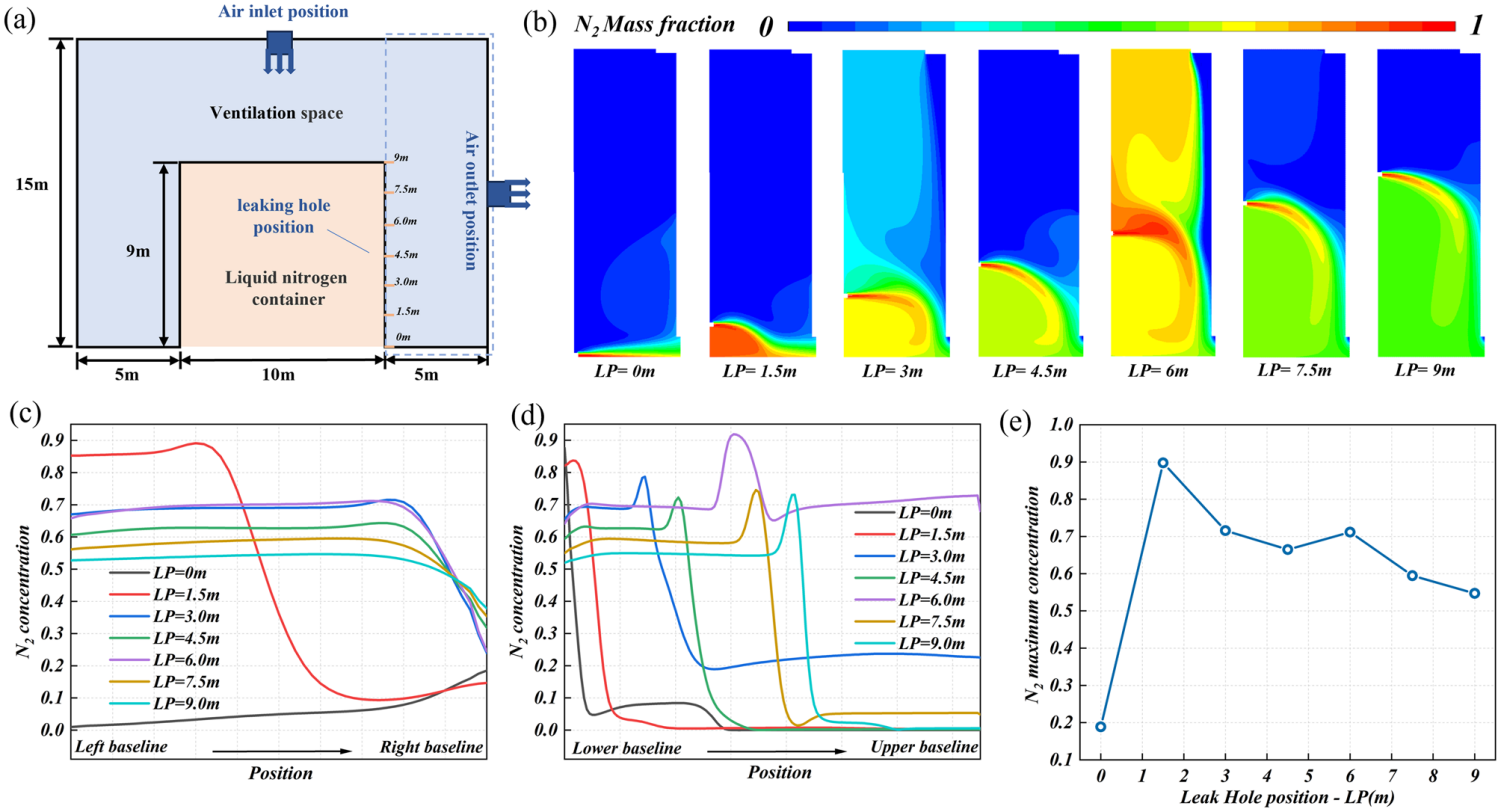
The nitrogen diffusion behavior at different potential leakage hole positions: (**a**) the work condition diagram, (**b**) the concentration distribution cloud diagram of leakage nitrogen in the research area, (**c**) the nitrogen concentration distribution on monitoring line 1, (**d**) the nitrogen concentration distribution on monitoring line 2, and (**e**) the maximum nitrogen concentration on monitoring line 1.

The influence of the design parameters of the ventilation system on nitrogen diffusion is explored, and the potential leakage factors of nitrogen should also be taken into account. Although the potential leakage factors are uncontrollable compared to the ventilation design factors, it is beneficial to the ventilation system design to comprehensively consider the ventilation design factor and the potential leakage factor. The ground is set to baseline LP = 0, as shown in Fig. 10a. The potential leakage hole position is parameterized as the distance from the baseline to explore the influence of the potential leakage position on the nitrogen diffusion. Figure 10b shows the nitrogen concentration distribution in the research area under different leakage hole positions. When LP = 0 and 1.5 m, the nitrogen distribution area is relatively concentrated. Due to the small relative distance between the leakage hole and the exhaust outlet, the nitrogen diffusion process is shortened, nitrogen is quickly discharged, and the nitrogen distribution area is small. With the further increase of the potential leakage hole height, the nitrogen distribution area becomes larger. It is worth noting that at LP = 3 and 6 m, the discharge of nitrogen is affected by the air curtain, which aggravates the nitrogen diffusion process and makes the nitrogen distribution area larger.

Figure 10c shows the nitrogen concentration distribution on monitoring line 1 under different leakage hole positions. Combined with Fig. 10b and c, it can be found that due to the relative position of monitoring line 1 and the leakage hole, the concentration distribution law at LP = 0 and 1.5 m is different from that at other leakage hole positions. When LP > 3 m, the nitrogen concentration distribution is similar. The difference is that the nitrogen concentration decreases from 72% at LP = 3 m to 55% at LP = 9 m, showing a decreasing trend (Fig. 10e). Figure 10d shows the nitrogen concentration distribution on monitoring line 2 under different leakage hole positions. There is no doubt that the nitrogen diffusion height increases with the increase of the leakage hole height, and the overall distribution area of nitrogen increases. Based on the analysis of nitrogen concentration value and nitrogen distribution area, shortening the nitrogen diffusion path is an important means to improve the effect of nitrogen removal.

#### Size of leakage hole

**Figure 11:**
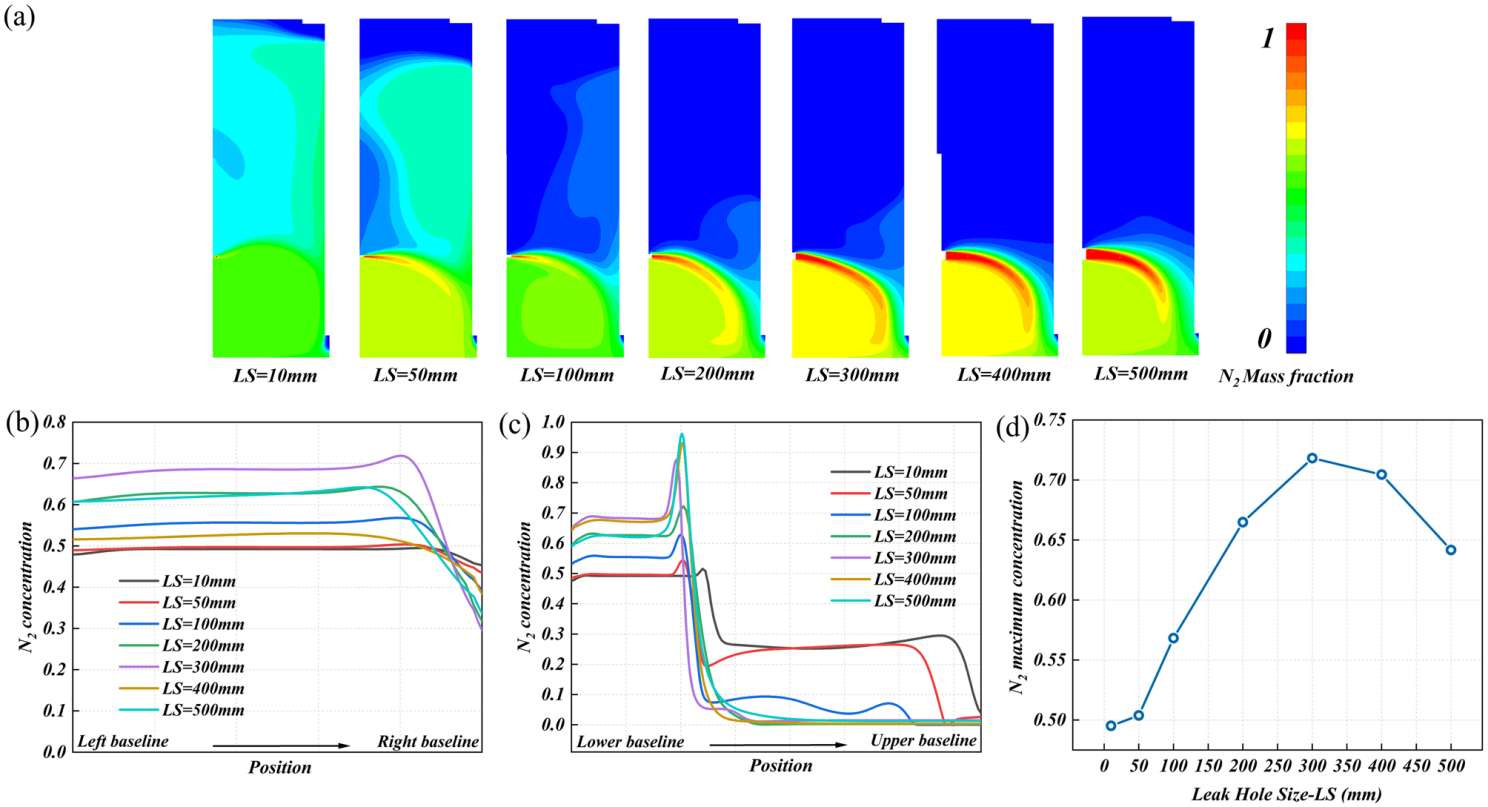
The nitrogen diffusion behavior at different potential leakage hole sizes: (**a**) the concentration distribution cloud diagram of leakage nitrogen in the research area, (**b**) the nitrogen concentration distribution on monitoring line 1, (**c**) the nitrogen concentration distribution on monitoring line 2, and (**d**) the maximum nitrogen concentration on monitoring line 1.

Figure 11a shows the concentration distribution of leakage nitrogen in the research area under different leakage hole sizes. It can be seen that under the small leakage hole size, the leaked nitrogen cannot break through the air curtain formed by the fresh air flow, resulting in a wider diffusion range. With the increase of the leakage hole size, the ‘breakthrough’ effect is enhanced, and the distribution area of the leakage nitrogen is concentrated and discharged under the carrying of the fresh air. Therefore, this also highlights the importance of fresh air velocity design.

Figure 11b and c are the nitrogen concentration distribution on monitoring line 1 and monitoring line 2 under different leakage hole sizes. It can be seen from Fig. 11b that the larger the leakage hole size, the earlier the concentration attenuation near the right baseline on the monitoring line 1, and the more obvious the attenuation effect. The maximum concentration on monitoring line 1 is positively correlated with the leakage hole size. It increased from 48% at LS = 10 mm to 72% at LS = 300 mm (Fig. 11d). It can be seen from Fig. 11c that when LS = 10, 50, and 100 mm, the nitrogen concentration fluctuates greatly after the first sudden decrease and does not drop to 0. It shows that nitrogen has a high diffusion height, especially at LS = 10 mm. When LS > 100 mm, the nitrogen concentration did not fluctuate and dropped to 0 after the first sudden decrease. It shows that the nitrogen distribution height is low, mainly concentrated in the lower part of the research area. The maximum concentration on monitoring line 2 is also positively correlated with the leakage hole size, from 51% at LS = 10 mm to 95% at LS = 500 mm. The leakage hole size has a crucial impact on the nitrogen removal performance, which requires regular maintenance work to prevent the occurrence of large leakage holes.

#### Mass flow rate of leaked nitrogen

**Figure 12:**
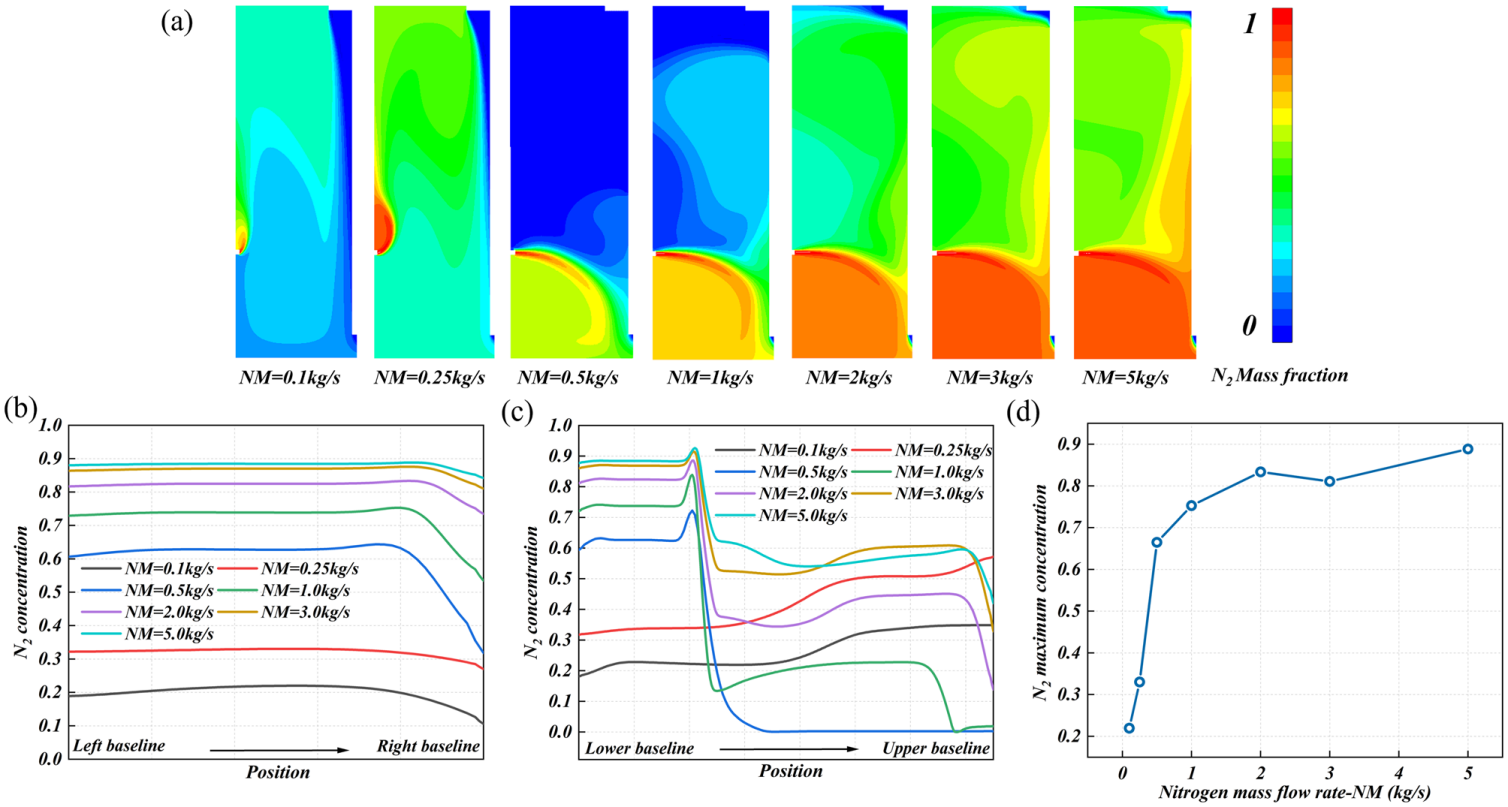
The nitrogen diffusion behavior at different mass flow rate of leaked nitrogen: (**a**) the concentration distribution cloud diagram of leakage nitrogen in the research area, (**b**) the nitrogen concentration distribution on monitoring line 1, (**c**) the nitrogen concentration distribution on monitoring line 2, and (**d**) the maximum nitrogen concentration on monitoring line 1.

Figure 12a shows the concentration distribution of leaked nitrogen in the research area under different mass flow rates of nitrogen. Under a low mass flow rate (NM = 0.1 and 0.25 kg/s), the leaked nitrogen cannot break through the air curtain formed by the fresh air flow. In this case, although the nitrogen concentration is low, its distribution area is large. When the mass flow rate is NM = 0.5 kg/s, the negative impact of this air curtain is weakened and it is in an ideal state of nitrogen removal. When the mass flow rate NM > 0.5 kg/s, the nitrogen removal performance of the ventilation system gradually disappears. In this case, with the increase of mass flow rate, the nitrogen concentration distribution is almost unchanged, and the nitrogen diffuses to the whole research space.

Figure 12b and c are the nitrogen concentration distribution on monitoring line 1 and monitoring line 2 under different nitrogen mass flow rates. It can be seen from Fig. 12b that when NM = 0.1 and 0.25 kg/s, the nitrogen concentration is almost not fluctuating, which is the corresponding phenomenon that the leaked nitrogen cannot break through the fresh air curtain. When the mass flow rate increased to 0.5 kg/s, the concentration distribution changed, and the concentration decreased sharply in the area near the right baseline. This sudden decrease gradually disappears with the increase of nitrogen mass flow rate, and finally tends to a straight line without fluctuation. The maximum nitrogen concentration on monitoring line 1 increased significantly with the increase of mass flow rate, from 22% at NM = 0.1 kg/s to 89% at NM = 5 kg/s. It is worth noting that when NM > 2 kg/s, the nitrogen removal performance of fresh air is abolished, and the nitrogen concentration is gradually stabilized (Fig. 12d). From Fig. 12c, it can be seen that only when NM = 0.5 kg/s, the concentration on monitoring line 2 is only suddenly reduced to 0 at the lower part, and the remaining cases are not suddenly reduced to 0, and there are great fluctuations, and the nitrogen distribution area is large.

## Prediction of nitrogen concentration and diffusion characteristics based on parametric analysis and GA-BP neural network

### Construction of the BP neural network

**Figure 13:**
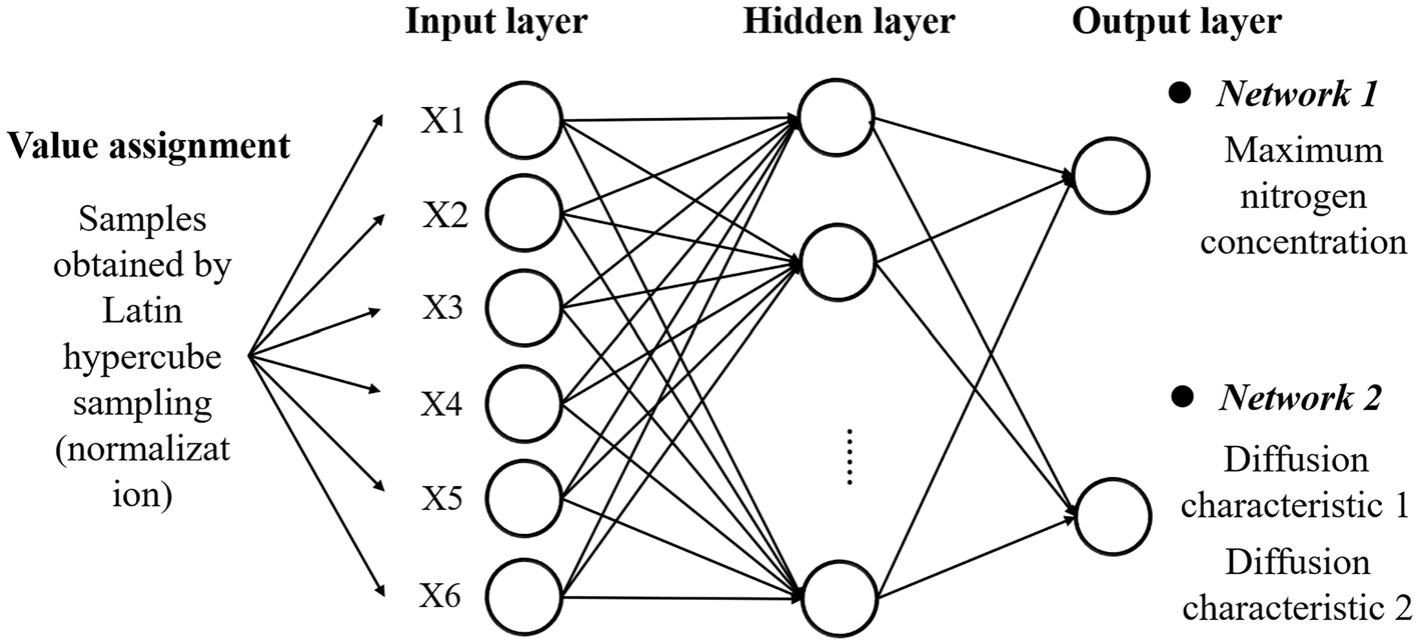
The neural network structure of numerical regression of nitrogen concentration and classification regression of nitrogen diffusion characteristics.

From the perspective of statistical analysis, when there are enough pairs of input and output variables, the nitrogen concentration and nitrogen diffusion characteristics under ventilation can be further expressed as a regression problem^50^. Through the analysis in section “Parametric study on the influencing factors of nitrogen diffusion behavior”, it can be seen that the evaluation of the nitrogen removal performance of the ventilation system should consider not only the nitrogen concentration but also the nitrogen diffusion characteristics. The nitrogen concentration can be measured by a numerical value. The nitrogen diffusion characteristics are divided into two categories. Type 1 is that nitrogen only diffuses in the ideal range surrounded by fresh air flow and environmental structure and then discharges. Type 2 is that part of nitrogen escapes from the ideal range surrounded by fresh air flow and environmental structure during the discharge process, and diverges and diffuses. The prediction of nitrogen concentration is a numerical regression problem, and the prediction of nitrogen diffusion characteristics is a classification regression problem. The fresh air inlet position (X1), fresh air velocity (X2), exhaust outlet position (X3), leakage position (X4), leakage hole diameter (X5), and leakage nitrogen mass flow rate(X6) are selected as input parameters. The numerical regression network 1 uses the maximum nitrogen concentration on monitoring line 1 as the output parameter, while the classification regression network 2 uses the nitrogen diffusion characteristics as the output parameter. The numerical regression neural network structure for predicting nitrogen concentration and the classification regression neural network structure for predicting nitrogen diffusion characteristics are shown in Fig. 13.

The structure of network 1 is 6-*c*-1, while the structure of network 2 is 6-* c* -2. The number of nodes in the hidden layer *c* has an important influence on the prediction accuracy. Too many nodes are prone to overfitting, and too few nodes will increase the error. Usually, the approximate interval of *c* is determined by Eq. (1)^51^, and the optimal number of *c* is found by trial and error method. By comparing the number of hidden layer nodes *c* of network 1 and network 2, the final structure of network 1 is determined to be 6-11-1, and the final structure of network 2 is 6-7-1.

1 $$c = \sqrt {(a + b)} + c_{0}$$

where $c$ is the number of hidden layer nodes, $a$ is the number of input layer nodes, $b$ is the number of output layer nodes, and $c_{0}$ is the constant of [0,10].

The fitness function is the main basis of the GA evolutionary search^52^. The fitness value $F_{fitness}$ used in this work is the sum of the absolute value of the error of the predicted values of all nodes in the output layer, as shown in Eq. (2).

2 $$F_{fitness} = \sum\limits_{m = 1}^{N} {\left( {\left| {O_{m} - P_{m} } \right|} \right)}$$

where *N* is the number of samples, $P_{m}$ and $O_{m}$ is the predicted value and the true value of the nitrogen concentration value, respectively.

The chromosomes in the GA part are encoded in real numbers, network 1 is $6 \times 11 + 11 \times 1{ + }11{ + }1{ = }89$ and network 2 is $6 \times 7 + 7 \times 2{ + }7{ + }2{ = }65$. The population range is 10, the maximum genetic algebra is 100, the selection operation uses the roulette method, the crossover probability is 0.7, and the mutation probability is 0.1. The hidden layer transfer function in the neural network part is the hyperbolic tangent Tansig function, the output layer function is the linear Purelin function, the learning rate is 0.01, the minimum training error is 1 × 10^–6^, and the training times are 1000.

### Sample data processing

#### Samples obtained by Latin hypercube sampling

The Latin hypercube sampling (LHS) is used in this work. LHS is a method of approximate random sampling from multivariate parameter distribution, which avoids more redundancy in the sample while ensuring the randomness of the sample^53^. The main idea is to stratify the probability distribution to reconstruct the probability distribution with fewer samples. Using a small number of sampling times can obtain the same results as a large number of random samples. The sampling range is uniform and there is no obvious aggregation phenomenon. Assuming that there are N variables, each variable can be divided into M intervals with the same probability. At this time, M samples that satisfy the Latin hypercube condition can be selected. Note that LHS requires the same number of partitions M for each variable. And when the number of variables increases, the specified number of samples M does not change. Forced extraction of samples from each partition ensures full coverage within a variable range, reflecting the comprehensiveness of the sample results. Compared with simple random sampling, the sample will not have the problem of excessive aggregation of sample data under the premise of comprehensiveness and representativeness. This method is especially suitable for the case of many variables and complex sampling environments. The LHS is performed on six factors according to the specified range: fresh air inlet position-IP [− 5,15], fresh air velocity-IV [0.1,5], exhaust outlet position-OP [0.5,14.5], leakage hole position-LP [0,9], leakage hole size-LS [10,500], and nitrogen mass flow rate-NM [0.1,5]. The samples obtained by LHS and the results obtained by CFD numerical simulation are shown in Table 7.

**Table 7:** The samples extracted by LHS and the results calculated by numerical simulation.

No.	Ventilation design factor			Potential leakage factor			Measure index	
	X1–IP (m)	X2–IV (m/s)	X3–OP (m)	X4–LP (m)	X5–LS (mm)	X6–NM (kg/s)	Maximum nitrogen concentration	Diffusion characteristics
1	10.60	1.16	9.56	3.96	355.13	0.81	0.9437173	1
2	2.93	1.41	12.44	1.97	91.40	4.94	0.8761185	2
3	13.70	0.31	6.94	8.83	392.10	1.73	0.9595441	2
4	7.64	3.38	8.28	3.42	60.54	2.16	0.6773743	1
5	4.57	1.80	1.16	2.94	79.36	4.69	0.7605445	1
6	9.53	3.02	3.69	0.94	316.04	3.16	0.9326984	1
7	12.04	3.63	10.47	3.14	185.98	4.45	0.9905429	1
8	14.21	2.69	6.62	0.20	339.49	2.76	0.9905429	1
9	− 4.76	2.88	13.37	4.34	414.70	2.31	0.9960957	2
10	11.55	2.45	2.90	7.09	287.05	0.22	0.1247146	1
11	5.34	0.80	14.17	5.74	409.60	3.94	0.9922832	1
12	14.85	2.57	9.26	1.76	144.56	3.01	0.8225862	1
13	2.14	4.20	11.04	1.41	395.40	3.08	0.9754507	1
14	0.30	4.46	14.26	7.37	495.32	3.53	0.9831423	1
15	9.05	1.73	5.61	8.62	474.29	0.34	0.3418074	1
16	− 0.74	1.61	7.62	4.75	17.48	2.52	0.6257722	2
17	1.53	3.10	8.57	2.17	194.46	4.55	0.8771484	1
18	12.88	3.49	11.71	8.22	207.67	4.08	0.8897126	1
19	3.19	2.34	12.19	3.71	365.08	0.91	0.9627872	1
20	10.27	1.54	2.20	2.70	294.84	1.14	0.8382359	1
21	8.36	4.90	4.08	6.85	469.54	0.54	0.1558133	1
22	4.87	0.60	7.40	0.06	204.21	0.13	0.8332691	1
23	− 0.32	0.72	1.60	0.36	126.63	2.85	0.8651513	2
24	7.22	0.99	9.00	5.80	24.80	3.89	0.8048891	2
25	− 3.17	1.26	6.89	5.34	439.31	4.33	0.9952348	1
26	6.06	3.24	3.57	4.56	109.02	3.26	0.7055951	1
27	6.78	3.16	4.78	6.44	224.63	0.69	0.3385827	1
28	7.90	0.44	13.56	6.25	283.97	3.82	0.972907	2
29	12.43	4.56	10.68	7.62	131.94	1.05	0.5676447	1
30	14.03	3.80	8.79	2.60	325.83	1.21	0.9772716	1
31	8.95	3.85	13.76	6.05	261.74	0.66	0.9533893	1
32	4.19	0.57	2.73	5.11	106.25	1.35	0.6998722	2
33	13.28	4.41	11.45	3.82	447.68	1.50	0.994313	1
34	3.67	4.63	11.20	0.79	47.14	1.80	0.5766313	1
35	− 4.46	4.93	9.81	7.46	171.60	2.69	0.8042774	2
36	− 2.98	4.24	4.19	3.49	148.12	2.58	0.8783501	2
37	11.03	3.96	10.19	5.49	268.44	2.06	0.7785242	1
38	0.82	2.80	5.93	6.83	38.40	2.12	0.5934371	1
39	10.10	1.34	3.14	2.03	231.57	4.76	0.9303482	1
40	− 1.31	2.18	5.11	2.34	58.46	4.19	0.7671219	2
41	2.30	0.95	12.74	4.98	73.69	3.36	0.8797911	2
42	6.30	0.22	1.83	4.15	456.57	1.58	0.9656451	1
43	− 4.10	4.73	5.41	8.40	157.02	1.93	0.5662892	1
44	5.76	1.98	1.00	7.94	305.85	0.42	0.2753321	1
45	− 0.07	1.91	7.83	6.58	485.44	4.89	0.9896209	1
46	− 3.59	2.10	0.50	0.65	236.43	4.23	0.08960952	1
47	1.32	2.46	13.09	1.22	381.05	3.52	0.9999993	1
48	− 1.84	3.58	4.55	1.60	427.58	2.39	0.9828585	2
49	− 2.53	0.19	2.16	7.86	245.91	1.44	0.8814598	2
50	− 1.47	4.03	6.16	8.69	344.01	3.65	0.890788	2

#### Data normalization processing

The neural network determines the network mapping relationship by learning the sample data. The sample data used for training and testing has a direct impact on the final performance of the network model. Different ranges of sample data will lead to large fluctuations in the results obtained by the neural network model during learning and training, which will affect the prediction ability of the model. Therefore, the sample data should be normalized before use^54^. At present, the most commonly used normalization method is the maximum and minimum method, as shown in Eq. (3)^55^.

3 $$X_{m}^{\prime } = \frac{{X_{m} - X_{\min } }}{{X_{\max } - X_{\min } }}$$

where $X_{m}$ and $X_{m}^{\prime }$ are the data before and after normalization, $X_{\max }$ and $X_{\min }$ are the maximum and minimum values of the sample data.

### Prediction of nitrogen concentration under ventilation condition

The samples obtained by LHS are disrupted to ensure the randomness of the samples. Considering the size of the sample space, the out-of-order and normalized data sets are divided into training sets and test sets by a ratio of 7: 3, and 35 training samples and 15 test samples are obtained. The training set and test set are imported into the GA-BP neural network for training and prediction.

**Figure 14:**
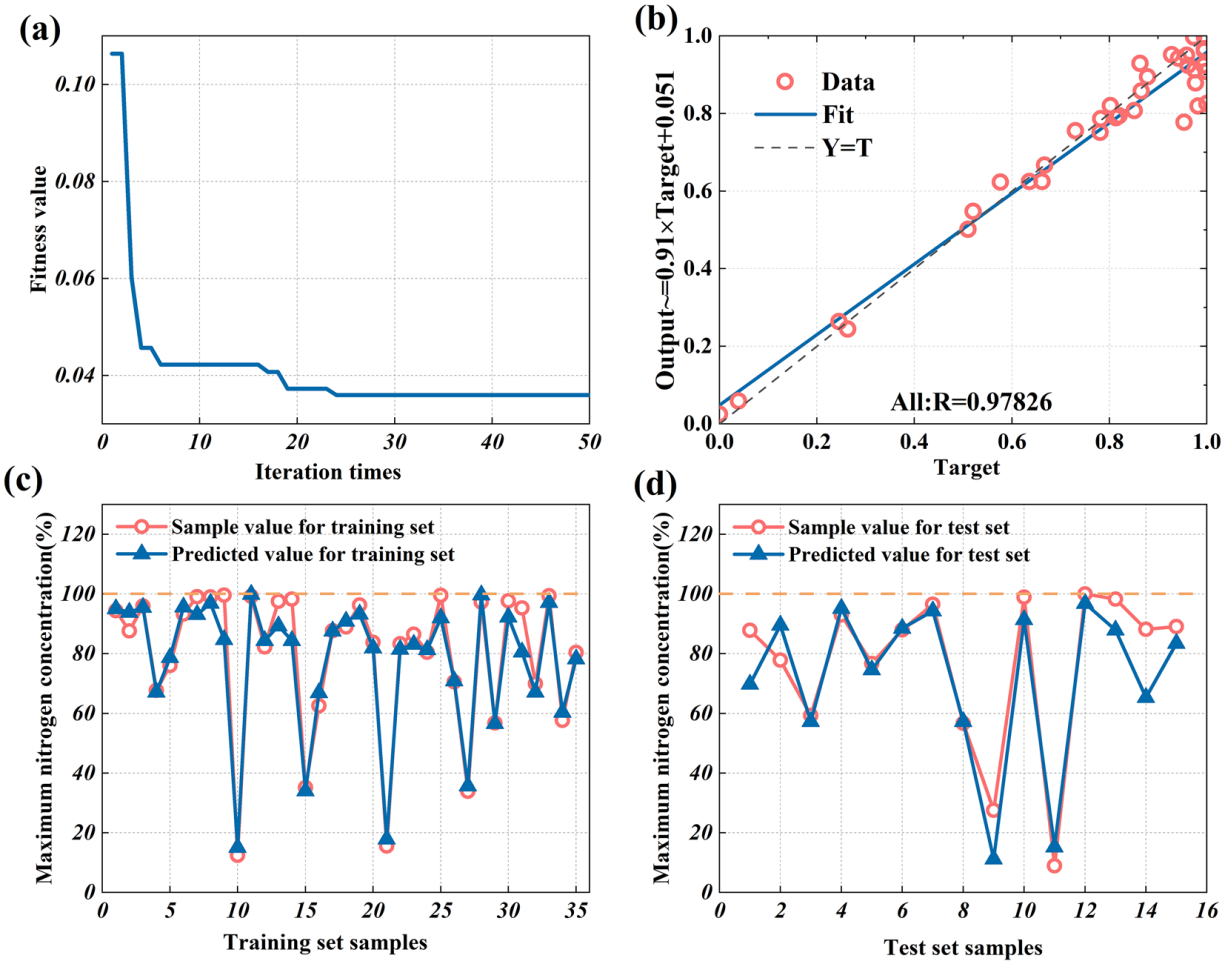
GA-BP neural network analysis for numerical regression prediction: (**a**) fitness curve, (**b**) the correlation between the actual output data and expected output data, (**c**) the comparison between the actual value and predicted value of the training set, (**d**) the comparison between the actual value and predicted value of the test set.

Figure 14a is the fitness evolution of the GA-BP neural network in predicting nitrogen concentration. In the iterative process, the fitness value shows a decreasing trend with the increase in the number of iterations, indicating that the GA optimization is evolving in the right direction. When the iteration is about 23 times, the fitness value tends to be stable, indicating that the weights and thresholds in the individual after 23 rounds of evolution have reached the optimal value. Figure 14b shows the correlation between the actual output data and the expected output data of samples. The correlation can be expressed by the correlation coefficient R. The absolute value range of R is [0,1]. The closer the absolute value of R is to 1, the stronger the linear correlation between the actual output data and the expected output data is. The correlation coefficient R between the actual output data and the expected output data of samples is 0.97826, which proves that the established GA-BP neural network has good approximation ability.

Figure 14c and d are the comparison between the actual value and the predicted value of the training set and the comparison between the actual value and the predicted value of the test set, respectively. It can be found that the predicted value and the actual value are in good agreement. The coefficient of determination (R^2^), mean absolute error (MAE), and root mean squared error (RMSE) are used as the evaluation indexes of model performance. The calculation equations are shown in Eqs. (4)–(6). These three evaluation indexes are widely used, among which the Coefficient of Determination (R^2^) is a statistical index used to evaluate the fit goodness of the regression model, which can represent the proportion of the variability of the dependent variable that can be explained by the model, that is, the fitting degree of the model to the data. The value range of R^2^ is [0, 1], and the higher R^2^ value indicates the better fitting degree of the model. Mean absolute error (MAE) and root mean square error (RMSE) are both indicators to measure the difference between the predicted value and the actual observed value of the model, which are used to evaluate the fitting degree of the model on the given data. The smaller MAE value and RMSE value indicate that the difference between the predicted value of the model and the actual observed value is small, that is, the fitting degree of the model is better.

The R^2^ of the training set is 0.94942, the MAE is 3.6226, and the RMSE is 5.3005. The R^2^ of the test set is 0.85417, the MAE is 7.4472, and the RMSE is 10.0609. It can be found that these rating indicators of the training set and test set are good. Although the deviation of the test set is slightly larger than the training set, it is within the acceptable range. The GA-BP neural network can predict the nitrogen concentration with high accuracy, so it is effective to determine the nitrogen concentration according to the ventilation factors and potential leakage factors.

4 $$R^{2} = 1 - \frac{{\sum\limits_{m = 1}^{N} {\left( {P_{m} - O_{m} } \right)^{2} } }}{{\sum\limits_{m = 1}^{N} {\left( {\overline{{P_{m} }} - O_{m} } \right)^{2} } }}$$

5 $$RMSE = \sqrt {\frac{{\sum\limits_{m = 1}^{N} {\left( {P_{m} - O_{m} } \right)^{2} } }}{N}}$$

6 $$MAE = \frac{{\sum\limits_{m = 1}^{N} {\left| {P_{m} - O_{m} } \right|} }}{N}$$

where $\overline{{P_{m} }}$ is the predicted average value of the nitrogen concentration value.

### Prediction of nitrogen diffusion characteristics under ventilation condition

Similarly, the samples obtained by LHS are disrupted, and the out-of-order and normalized data sets are divided into the training set and test set in a ratio of 7: 3, and 35 training samples and 15 test samples are obtained. The sample data is input into the GA-BP neural network for training and prediction.

**Figure 15:**
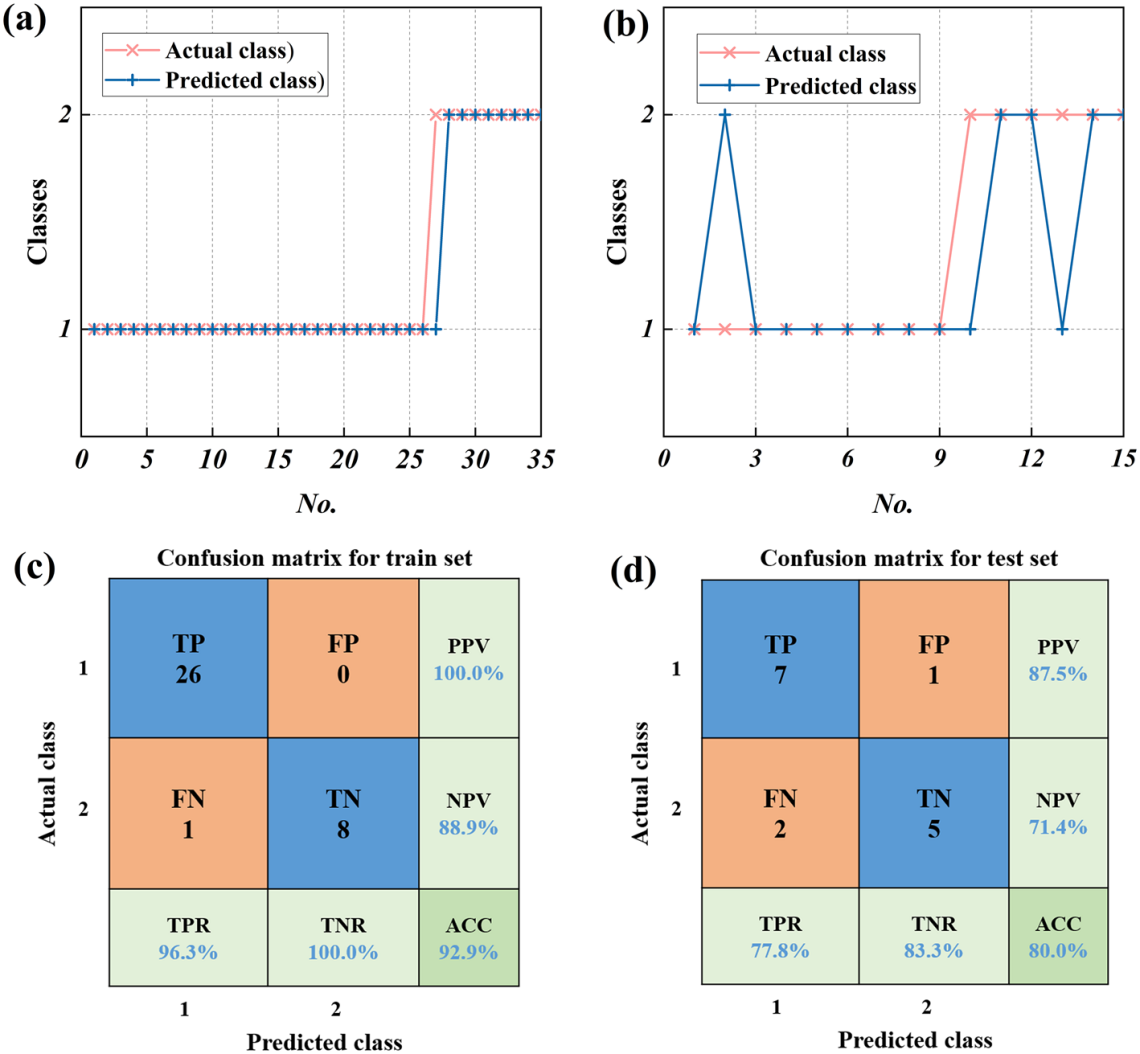
GA-BP neural network analysis for classification regression prediction: (**a**) comparison of actual classification and prediction classification of training set, (**b**) comparison of actual classification and prediction classification of test set, (**c**) statistics of training set classification results, and (**d**) statistics of test set classification results.

Figure 15a and b show the comparison of actual classification and predictive classification of the training set and the test set, respectively. There is only one prediction classification error in the training set, and diffusion characteristic 2 is judged as diffusion characteristic 1. There are three prediction classification errors in the test set. Among them, one group of diffusion characteristics 1 is identified as diffusion characteristics 2, and two groups of diffusion characteristics 2 are identified as diffusion characteristics 1. In order to further analyze the accuracy of the prediction model, the confusion matrix is used to evaluate the performance of the GA-BP neural network classification model. Since this work is a 2-classification problem, the confusion matrix is a 2 × 2 matrix, which can show the correspondence between the prediction results of the classification model and the actual situation. The four elements in the confusion matrix are respectively represented: True Positive (TP), False Positive (FP), True Negative (TN), and False Negative (FN), as shown in Fig. 15c and d. Accuracy (ACC), true positive rate (TPR), true negative rate (TNR), positive predictive value (PPV), and negative predictive value (NPV) are commonly used to measure. The equations are shown in Eqs. (7)–(11).

7 $$ACC = \frac{TP + TN}{{TP + TN + FP + FN}}$$

8 $$TPR = \frac{TP}{{TP + FN}}$$

9 $$TNR = \frac{TN}{{TN + FP}}$$

10 $$PPV = \frac{TP}{{TP + FP}}$$

11 $$NPV = \frac{TN}{{TN + FN}}$$

where TP, FP, TN, and FN represent their respective quantities.

Figure 15c and d show the classification confusion matrix of the training set and the test set respectively. Among the 35 groups of samples in the training set, there are 26 groups of diffusion characteristics 1 and 8 groups of diffusion characteristics 2. Only one group is misclassified. In the classification prediction of the training set, TPR is 96.3%, TNR is 100%, PPV is 100%, and NPV is 88.9%. The accuracy rate (ACC) can reach 92.9%. Among the 15 groups of samples in the training set, 7 groups are diffusion characteristics 1, and 5 groups are diffusion characteristics 2. Three groups are misclassified. In the classification prediction of the test set, TPR is 77.8%, TNR is 83.3%, PPV is 87.5%, and NPV is 71.4%. The accuracy rate (ACC) can reach 80%. The accuracy of the training set is slightly higher than that of the test set, but the overall accuracy is within the acceptable range. It is shown that the GA-BP neural network model can identify the nitrogen diffusion characteristics with high accuracy, so it is effective in determining the nitrogen diffusion characteristics according to ventilation factors and potential leakage factors.

## Conclusion

This work is based on the leakage and diffusion problem of large liquid nitrogen tanks in the China Jinping Underground Laboratory (CJPL). The influence of fresh air inlet position, fresh air velocity, exhaust outlet position, leakage hole position, leakage hole size, and nitrogen mass flow rate on nitrogen diffusion behavior and characteristics in a specified environment Is studied using the CFD method. The BP neural network optimized by GA is used to train the samples obtained from LHS, establishing a prediction model for nitrogen concentration and diffusion characteristics with good generalization ability and multi-factor and multi-range prediction ability. The conclusion is as follows:

1. In a deep laboratory without natural wind, good ventilation design is the main means to deal with the decrease in oxygen concentration caused by nitrogen leakage. When the fresh air inlet is located on the same side as the potential leakage hole, its nitrogen removal effect is better. When the fresh air inlet and the potential leakage hole are not on the same side, the fresh air flow will flow around and the flow field will change, which makes the nitrogen distribution area fluctuate greatly. The nitrogen concentration will gradually decrease as the fresh air inlet shifts towards the direction of the potential leakage hole, and the coverage of the fresh air curtain will increase, which is conducive to suppressing the divergent diffusion of nitrogen. The fresh air velocity and the nitrogen removal effect only show a positive correlation within an appropriate range of fresh air velocity. At low wind speeds, the nitrogen removal effect is not obvious. Under excessive wind speed, the fresh air curtain will block the exhaust outlet, hinder the nitrogen discharge, and lose the nitrogen discharge ability. The increase of the exhaust outlet height will increase the nitrogen diffusion distance, which makes the nitrogen diffusion area obviously diverge, and is not conducive to improving the nitrogen removal effect.
2. Compared with ventilation design factors, potential leakage factors are uncontrollable, but comprehensive consideration of ventilation design factors and potential leakage factors can provide a focus direction for safety protection. The nitrogen diffusion area will increase with the increase of the leakage hole height. Shortening the relative distance between the exhaust outlet and the leakage hole will help shorten the nitrogen diffusion path and inhibit the nitrogen divergence. Leakage hole size and nitrogen mass flow rate have similar effects on nitrogen diffusion behavior. Under a small leakage hole size and small nitrogen mass flow rate, the leaked nitrogen cannot break through the fresh air curtain, resulting in a wide nitrogen diffusion range. This situation will be improved with the increase of leakage hole size and nitrogen mass flow rate, and the nitrogen distribution area will gradually concentrate. The nitrogen concentration is positively correlated with the leakage hole size and the nitrogen mass flow rate. It is worth noting that this change only exists when the fresh air velocity is fixed. When the fresh air velocity does not match the leakage hole size and the nitrogen mass flow rate, the nitrogen removal capacity of the ventilation system is easily lost, and nitrogen will diffuse to the entire research space.
3. Nitrogen concentration and nitrogen diffusion characteristics should be considered when evaluating the nitrogen removal performance of the ventilation system. The nitrogen concentration is measured by numerical value, and the nitrogen diffusion characteristics are measured by category. The GA-BP neural network numerical regression and classification regression models for nitrogen concentration prediction and nitrogen diffusion characteristics prediction are established. Six influencing factors are parameterized, and LHS is used for uniform sampling within the specified range of each factor to obtain samples that can represent the entire sample space. Based on the samples obtained by LHS, the GA-BP neural networks are trained. Various rating indicators (R^2^, MAE, RMSE, ACC, TPR, TNR, PPV, and NPV) are used to evaluate the performance of the trained nitrogen concentration numerical prediction model and the nitrogen diffusion characteristic classification prediction model. The models have a high recognition rate and accuracy. It shows that it is very effective in predicting and determining the concentration and diffusion characteristics of nitrogen based on ventilation factors and potential leakage factors.

## Data Availability

All data generated or analysed during this study are included in this published article.

## List of symbols

$$x$$: Displacement (m)
$$u$$: Velocity (m/s)
$$E$$: Total energy (J)
$p$: Absolute pressure (Pa)
$$g$$: Gravitational acceleration (m/s^2^)
$$F$$: Vent area (m^2^)
$k$: Conductivity factor (W/mK)
$$T$$: Temperature (K)
$R$: Gas constant (J/kgK)
$M_{a}$: Relative molecular mass of air
$M_{v}$: Relative molecular mass of nitrogen
$v$: Specifc volume (m^3^/kg)
$$C_{1\varepsilon }$$: Empirical constant
$$C_{2\varepsilon }$$: Empirical constant
$$C_{\mu }$$: Empirical constant
$G_{\kappa }$: Turbulence kinetic energy generation due to the mean velocity gradients
$G_{b}$: Turbulence kinetic energy generation due to buoyancy
$YM$: Effect of fluctuating expansion on total dissipation rate in compressible turbulence
$\Pr_{t}$: Turbulent Prandtl number
$$I$$: Turbulence intensity
$a$: Number of neurons in input layer
$c$: Number of hidden layer neurons
$b$: Number of neurons in the output layer
$A_{m}$: The input of the $m$th hidden layer neuron
$x_{m}$: Vector values of input layer
$b_{q}$: Threshold of hidden layer neurons
$Y_{m}$: The output of the $m$th hidden layer neuron
$f_{1}$: Transfer function from input layer to hidden layer
$S_{q}$: The input value of the $q$th output layer
$a_{q}$: Threshold of output layer neurons
$Q_{q}$: The output of the qth output layer neuron
$f_{2}$: Transfer function from hidden layer to output layer
$MSE$: Mean square error
$P_{q}$: The expected output value of the $q$th neuron
$N$: Total number of training samples
$$c_{0}$$: Constant of [0,10]
$O_{m}$: Original value of the nitrogen concentration value
$P_{m}$: Predicted value of the nitrogen concentration value
$\overline{{P_{m} }}$: Predicted average value of the nitrogen concentration value
$X_{m}$: Data before normalization
$X_{m}^{\prime }$: Data after normalization
$X_{\max }$: Maximum values of the sample data.
$X_{\min }$: Minimum values of the sample data.
$R^{2}$: Coefficient of determination
$RMSE$: Root mean square error
$MAE$: Mean absolute error
$$TP$$: True Positive
$$TN$$: True Negative
$$FP$$: False Positive
$$FN$$: False Negative
$$ACC$$: Accuracy
$TPR$: True positive rate
$TNR$: True negative rate
$PPV$: Positive predictive value
$NPV$: Negative predictive value
CFD: Computational fluid dynamics
ANN: Artificial neural network
GA: Genetic algorithm
CJPL: China Jinping Underground Laboratory
LHS: Latin hypercube sampling
BP: Backpropagation
